# Tumour suppressor 15-hydroxyprostaglandin dehydrogenase induces differentiation in colon cancer via GLI1 inhibition

**DOI:** 10.1038/s41389-020-00256-0

**Published:** 2020-08-19

**Authors:** Shakti Ranjan Satapathy, Geriolda Topi, Janina Osman, Karin Hellman, Fredrik Ek, Roger Olsson, Wondossen Sime, Lubna M. Mehdawi, Anita Sjölander

**Affiliations:** 1 Cell and Experimental Pathology, Department of Translational Medicine, Lund University, Skåne University Hospital, Malmö, Sweden; 2 Chemical Biology & Therapeutics Group, Department of Experimental Medical Science, Lund University, Lund, Sweden; 3 Present Address: Department of Laboratory Medicine, Translational Cancer Research, Lund University, Medicon Village, Lund, Sweden

**Keywords:** Colorectal cancer, Cancer microenvironment

## Abstract

Inflammation is an established risk factor for colorectal cancer. We and others have shown that colorectal cancer patients with elevated cysteinyl leukotriene receptor 2 (CysLT_2_R) and 15-hydroxyprostaglandin dehydrogenase (15-PGDH) levels exhibit good prognoses. However, both CysLT_2_R and 15-PGDH, which act as tumour suppressors, are often suppressed in colorectal cancer. We previously reported that leukotriene C_4_ (LTC_4_)-induced differentiation in colon cancer via CysLT_2_R signalling. Here, we investigated the involvement of Hedgehog (Hh)–GLI1 signalling, which is often hyperactivated in colorectal cancer. We found that the majority of colorectal cancer patients had high-GLI1 expression, which was negatively correlated with CysLT_2_R, 15-PGDH, and Mucin-2 and overall survival compared with the low-GLI1 group. LTC_4_-induced 15-PGDH downregulated both the mRNA and protein expression of GLI1 in a protein kinase A (PKA)-dependent manner. Interestingly, the LTC_4_-induced increase in differentiation markers and reduction in Wnt targets remained unaltered in *GLI1*-knockdown cells. The restoration of GLI1 in *15-PGDH*-knockdown cells did not ameliorate the LTC_4_-induced effects, indicating the importance of both 15-PGDH and GLI1. LTC_4_-mediated reduction in the DCLK1 and LGR5 stemness markers in colonospheres was abolished in cells lacking 15-PGDH or GLI1. Both DCLK1 and LGR5 were highly increased in tumour tissue compared with the matched controls. Reduced Mucin-2 levels were observed both in zebrafish xenografts with *GLI1*-knockdown cells and in the *cysltr2*^−/−^ colitis-associated colon cancer (CAC) mouse model. Furthermore, GLI1 expression was positively correlated with stemness and negatively correlated with differentiation in CRC patients when comparing tumour and mucosal tissues. In conclusion, restoring 15-PGDH expression via CysLT_2_R activation might benefit colorectal cancer patients.

## Introduction

Colorectal cancer (CRC), one of the most prevalent cancers in the world, has a high metastatic efficacy and a low 5-year survival rate^1^. A nontargeted therapeutic approach combined with late diagnosis leads to poor prognosis and treatment failure. More than 85% of CRC cases exhibit anomalous APC/Wnt/β-catenin signalling, which regulates the progression of CRC by adopting different intracellular mechanisms, thus affecting cancer stem cells and interactions with the tumour microenvironment. Hedgehog (Hh) signalling, which regulates differentiation under physiological conditions, has attracted attention because of its emerging role in the promotion and maintenance of CRC^2–4^. In the untransformed colon, Hh ligands are secreted by epithelial cells targeting mesenchymal cells as a classic paracrine Hh signalling pathway to ensuring the proper size and location of the crypt–villus axis^5^, as also observed in other tissues^6^.

In CRC, abnormal Hh signalling functions in a ligand-dependent manner and is activated in human CC cell lines^7^ and xenograft models^4^. However, the role of Hh signalling and its importance in cell survival in CRC are not well defined. Although some previous studies have failed to derive a positive correlation between Hh signalling and CRC initiation and maintenance^8,9^, major bodies of evidence point to a positive correlation^3,4,7^. Moreover, previous reports have suggested high activity of the Hh–SMO–GLI axis in CRC cell survival and metastasis, which is coordinated by either canonical signalling (via SMO) or a non-canonical mode of activation (via the RAS-MAP kinase pathway)^4,7^. Within these pathways, the most prominent factors are glioma-associated oncogene homologue (GLI) 1 and 2 and the transcriptional regulators downstream of SMO, which keep the oncogenic pathway active.

CRC, which is considered to be an inflammation-associated cancer, is greatly influenced by inflammatory mediators, such as leukotrienes and prostaglandins, which belong to the G-protein-coupled receptor family. The cysteinyl leukotriene receptors (CysLTRs) CysLT_1_R and CysLT_2_R are activated by binding with their high-affinity ligands leukotriene D_4_ and C_4_ (LTC_4_), respectively^10,11^. These proinflammatory lipid mediators are derived from the arachidonic acid pathway via 5-lipoxygenase, and they play crucial roles in pathological inflammation, such as that observed in inflammatory bowel disease. We previously showed that elevated CysLT_1_R levels were associated with poor prognoses in CRC patients, while patients with high CysLT_2_R expression had better prognoses^12^. Another important group of eicosanoids are prostaglandins (PGs), which are produced via the COX-2 pathway. The upregulation of COX-2 in CRC increases the PGE_2_ level, which promotes cancer cell proliferation, angiogenesis, survival, migration, and invasion; these are important hallmarks of cancer^13,14^.

The tumour suppressor 15-hydroxyprostaglandin dehydrogenase (15-PGDH) is an enzyme responsible for the degradation of PGE_2_ into an inactive metabolite^15^. 15-PGDH is abundantly expressed in normal colon mucosa, but its expression is lost in CRC cells^16,17^, leading to disease progression. Recently, researchers have explored the efficacy of 15-PGDH as a potential antitumour agent against colon cancer^18–21^. In a recent study, we established that 15-PGDH is induced by LTC_4_ via CysLT_2_R signalling by phosphorylating c-Jun N-terminal kinase and AP-1 to induce 15-PGDH promoter activity and further guide colon cancer cells toward redifferentiation^20^. However, the detailed mechanism underlying this phenomenon remains unclear.

In this study, we elucidated the mechanism by which LTC_4_-induced 15-PGDH promotes differentiation in colon cancer cells through CysLT_2_R activation with the involvement of Hh–GLI signalling. We observed that GLI1 was involved in the regulation of the redifferentiation and reduction in stemness induced by LTC_4_ via 15-PGDH in colon cancer cells.

## Results

### GLI1 expression is negatively correlated with CysLT2R, 15-PGDH, and Mucin-2 expression in CRC patients

**Fig. 1:**
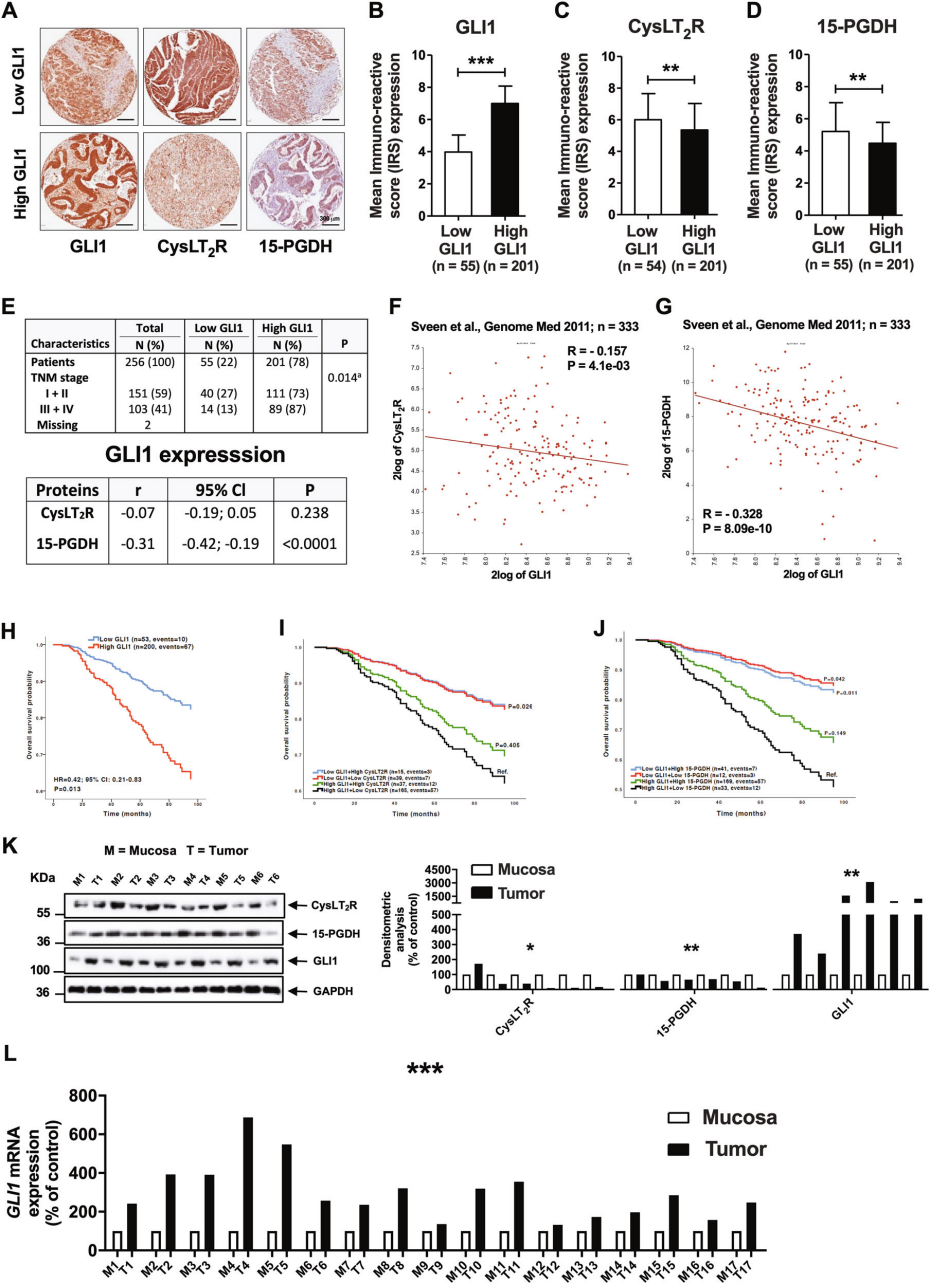
GLI1 expression exhibited a negative correlation with CysLT_2_R and 15-PGDH expression in colorectal cancer patient tissues. **a** Matched pair immunohistochemistry (IHC) images of GLI1, CysLT_2_R, and 15-PGDH expression in patients with low- and high-GLI1 expression, shown at ×20 magnification. The staining immunoreactivity was quantified by the mean immunoreactive score (IRS), calculated according to the following formula: IRS = (staining intensity) × (% of stained cells). The mean IRS for the groups of patients with low- and high-GLI1 expression. **b** The *Y*-axis represents IRS for GLI1 in these patients. **c** CysLT_2_R and **d** 15-PGDH expression according to patients with low- and high-GLI1 expression in CRC tissue. **e** Distribution of tumour-node-metastasis (TNM) stages of CRC according to low- and high-GLI1 expression. Pairwise Pearson correlation coefficient (*r*) between the expression of GLI1 and that of CysLT_2_R and 15-PGDH. *P* value according to chi-square test. *XY*-scatter plots showing mRNA levels of **f** *GLI1* and *CysLT*_*2*_*R* and **g** *GLI1* and *15-PGDH* (*HPGD*) gene expression from a public database containing 333 CRC patients. Kaplan–Meier curves for overall survival adjusted for age and TNM stage for patients with **h** low- and high-GLI1 expression and subgroups of patients with both GLI1 and **i** CysLT_2_R or **j** 15-PGDH expression compared by the log-rank test. The last patient group served as the reference category. **k** Western blot analysis showing the protein expression of CysLT_2_R, 15-PGDH and GLI1 in matched pairs of six patients with normal (N) and tumour (T) areas. KDa indicated on the left side of the immunoblots (KDa 55, 36, 100, 36) represents protein size markers. Graphical representation of the densitometric analysis showing the relative protein expression for CysLT_2_R, 15-PGDH, and GLI1 in matched pairs of patient samples (*n* = 6) from normal mucosa (M, white) and tumour (T, black) areas. **l** qRT-PCR analysis of *GLI1* in matched pairs of normal mucosa (M, white) and tumour (T, black) tissues from CRC patients (*n* = 17). Scale bar as indicated in the images. Data represent the mean ± SD, *P < 0.05, **P < 0.01, ***P < 0.001, Mann–Whitney test.

To elucidate the regulatory activity of GLI1 on the antitumorigenic proteins CysLT_2_R and 15-PGDH and the differentiation marker Mucin-2 in colon cancer, we used a tissue microarray (TMA) of primary CRCs from 326 patients^22^. After IHC analysis, we found only five patients with negative GLI1 staining, 50 patients with weak staining intensity, 168 patients with moderate staining intensity, and 33 patients with strong staining intensity. The mean ± standard deviation (SD) of the immunoreactive score (IRS) for GLI1 expression was 6.3 ± 1.6. Then, we grouped the patients with negative and weak staining intensity and defined them as the low-GLI1 expression group (*n* = 55), and those with moderate and strong staining intensity were defined as the high-GLI1 expression group (*n* = 201; Fig. 1a). Seventy patients had missing or incomplete cores and were excluded from the final analysis.

After IHC evaluation of CysLT_2_R and 15-PGDH, we observed that patients with high-GLI1 expression had significantly lower levels of CysLT_2_R (IRS 5.4 ± 1.7) and 15-PGDH (IRS 4.5 ± 1.3) expression than those with low-GLI1 expression (Fig. 1b–d). Furthermore, there was a significant negative correlation between GLI1 and 15-PGDH (*r* = −0.31, *P* < 0.0001), suggesting hyperactivated Hh–GLI signalling with suppressed 15-PGDH (Fig. 1e). However, no significant correlation was found between GLI1 and CysLT_2_R expression (Fig. 1e).

On the other hand, patients with low-GLI1 expression had a significantly higher IRS for CysLT_2_R (6.0 ± 1.7) and 15-PGDH (5.2 ± 1.8) expression (Fig. 1c, d), which indicates the adverse effects of CysLT_2_R/15-PGDH axis activation on GLI1. We also noticed that the majority of patients exhibited high-GLI1 expression (201/256 patients), and when stratified according to the tumour-node-metastasis (TNM) staging, a significantly stronger association for the patients with TNM stages III and IV was found (Fig. 1e). In addition to the above observations, transcriptome data from a public database^23^ also suggested a significant negative correlation between *GLI1* and the tumour suppressors *CYSLTR2* (Fig. 1f) and *HPGD* (15-PGDH), (Fig. 1g).

Importantly, we observed that patients with low-GLI1 expression had a 58% lower risk of overall mortality (hazard ratio = 0.42; 95% CI, 0.21–0.83) than patients with high-GLI1 expression after adjusting for age and TNM stage (Fig. 1h; the unadjusted survival curve is provided in Supplementary Fig. S1A). The median follow-up time was 69.5 months (5.8 years), with 79 total events. This suggests a prominent role of GLI1 in colon carcinogenesis.

We have previously shown that CRC patients with low-CysLT_2_R and/or low-15-PGDH expression have a poor prognosis^12,20^. In this study, we noted that patients with high-GLI1 expression coupled with either low-CysLT_2_R expression or low-15-PGDH expression had poorer prognoses than patients with either low-GLI1 and low-15-PGDH expression or low-GLI1 and low-CysLT_2_R expression (Fig. 1i, j). Western blot analysis of six matched pairs of CRC patients showed significantly higher protein expression of CysLT_2_R and 15-PGDH in normal tissue than in matched tumour tissue (Fig. 1k). However, GLI1 showed elevated expression in tumour tissue compared with matched normal tissue (Fig. 1k).

**Fig. 2:**
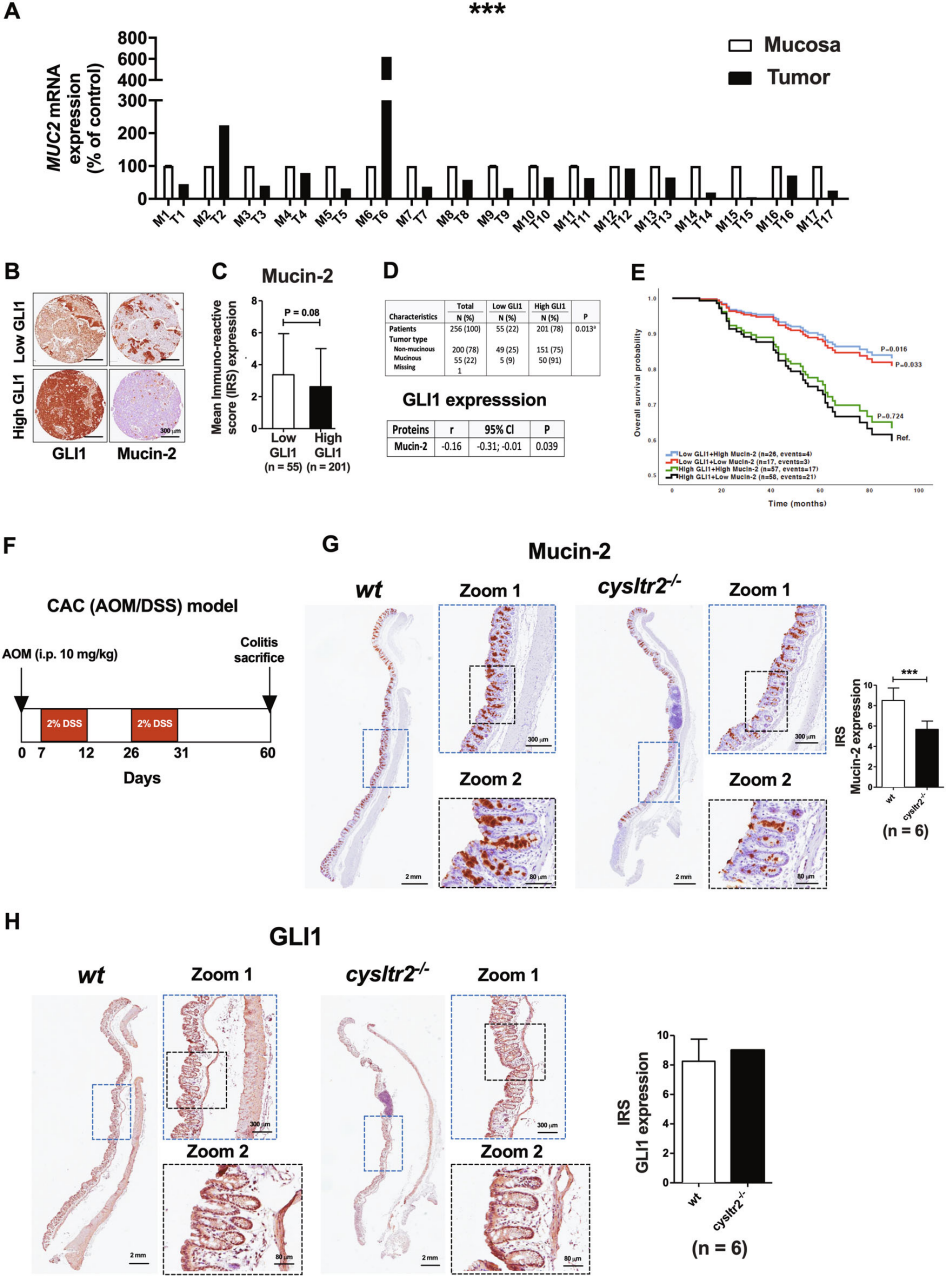
GLI1 expression was negatively correlated with differentiation. **a** qRT-PCR analysis of *MUCIN-2* in matched pairs of normal mucosa (M, white) and tumour (T, black) tissues from CRC patients (*n* = 17). **b** Immunohistochemistry (IHC) images for GLI1 and Mucin-2 expression in matched paired tissue samples from colorectal cancer (CRC) patients with low- and high-GLI1 expression represented at ×20 magnification. **c** Quantification of the staining immunoreactivity by the mean IRS for Mucin-2 expression according to patients with low- and high-GLI1 expression in CRC tissue. **d** Distribution of tumour type, mucinous adenocarcinomas versus non-mucinous adenocarcinomas, according to low- and high-GLI1 expression. Pairwise Pearson correlation coefficient (*r*) between the expression of GLI1 and Mucin-2. *P* value according to chi-square test. **e** Kaplan–Meier curves for overall survival adjusted for age and TNM stage subgroups of patients with both GLI1 and Mucin-2 expression compared by the log-rank test. The high-GLI1 and low-Mucin-2 patient group was set as the reference category. **f** Experimental schematic of the AOM–DSS mouse model. GLI1 expression exhibited a negative correlation with differentiation in the *cysltr2*^−/−^ AOM–DSS mouse model. Immunohistochemical evaluation showing the protein expression of **g** Mucin-2 and **h** GLI1 in *wt and cysltr2*^−/−^ AOM–DSS-challenged mice. Graph bars showing the IRS scores for Mucin-2 and GLI1, respectively, compared between *wt and cysltr2*^−/−^, *n* = 6 mice/group. Scale bar as indicated in the images. Data represent the mean ± SD, ***P < 0.001, Mann–Whitney test.

Furthermore, the mRNA analysis of these paired tumour tissues with matched mucosa tissues from CRC patients (*n* = 17) showed significantly higher mRNA expression of *GLI1* in the tumour tissue compared with its matched normal mucosa (the normal mucosa as reference set to 100 and tumour tissue 298.58 ± 35.69 (mean ± SEM), Mann–Whitney test, *P* < 0.001, Fig. 1l). Furthermore, the *MUC2* (MUCIN-2*)* mRNA analysis of these paired tumour tissues (T) and matched normal mucosa (M) showed significantly lower mRNA expression of *MUC2* in the tumour tissue compared with its matched normal mucosa (the normal mucosa as reference set to 100 and tumour tissue 92.60 ± 34.91 (mean ± SEM), Mann–Whitney test, *P* < 0.001, Fig. 2a). A representative matched pair of high- and low-GLI1 and the corresponding Mucin-2 is shown (Fig. 2b). We observed that patients with high-GLI1 expression had lower levels of Mucin-2 expression than those with low-GLI1 expression (Fig. 2c), and by combining these data, a significant negative correlation between elevated expression of GLI1 and decreased expression of Mucin-2 in tumour tissues was found (Fig. 2d). Moreover, grouping the patients into mucinous (*n* = 55) and non-mucinous (*n* = 200) types revealed that 75% of patients (151/200) with non-mucinous status had high-GLI1 expression, suggesting a negative correlation with Mucin-2-expressing cells, while 91% of patients (50/55) in the mucinous category showed high-GLI1 expression (Fig. 2d). Furthermore, we found a significant negative correlation between GLI1 and the differentiation marker Mucin-2 expression in these CRC patients (*n* = 158; Fig. 2e). We found a better overall survival for patients with low-GLI1 than those with high-GLI1 expression regardless of Mucin-2 expression (Fig. 2e). Taken together, these results suggest that GLI1 expression is negatively correlated with CysLT_2_R, 15-PGDH, and Mucin-2 expression but positively with the mucinous status of the patients.

### CysLT_2_R is essential for differentiation in a colitis-associated colon cancer—CAC—mouse model

To further validate the role of CysLT_2_R in promoting differentiation in CRC, we adopted an inflammatory mouse model that was induced by azoxymethane (AOM) and dextran sodium sulfate (DSS). Briefly, C57BL/6N wild-type and *cysltr2*^−/−^ mice were subjected to AOM and two 2% DSS cycles^24^ (Fig. 2f) as described in the Materials and Methods section. We found that all mice formed polyps in the colon regardless the phenotype but *cysltr2*^−/−^ mice (*n* = 13) developed significant larger polyps (≥1.5 mm, *P* = 0.0159) in the colon compared with their wild-type (*wt*; *n* = 11) littermates (Supplementary Fig. S1B, C). This result indicates that *cysltr2*^−/−^ mice develop a more progressive disease. Supplementary Fig. S1 shows a representative image of colon polyps (Fig. S1D) and a likewise representative Haemotoxylin and Eosin-stained image (Fig. S1E) showing pre-malignant areas, aberrant crypt foci and metaplasia areas from both *wt* and *cysltr2*^−/−^ mice colon.

Colon tissue sections from *wt* mice showed abundant Mucin-2-expressing cells compared with sections from *cysltr2*^−/−^ mice (5.7 ± 0.3, *n* = 6; Fig. 2g). GLI1 exhibited a slightly higher but non-significant overall expression in *cysltr2*^−/−^ mouse tissue sections compared with *wt* tissue sections (7.5 ± 0.6, *n* = 6; Fig. 2h). Taken together, the above observations encouraged us to further investigate Hh–GLI signalling using both in vitro and in vivo colon cancer model systems and to delineate the involvement of CysLT_2_R and 15-PGDH in promoting differentiation.

### LTC4-induced 15-PGDH downregulates GLI1 in colon cancer cells

**Fig. 3:**
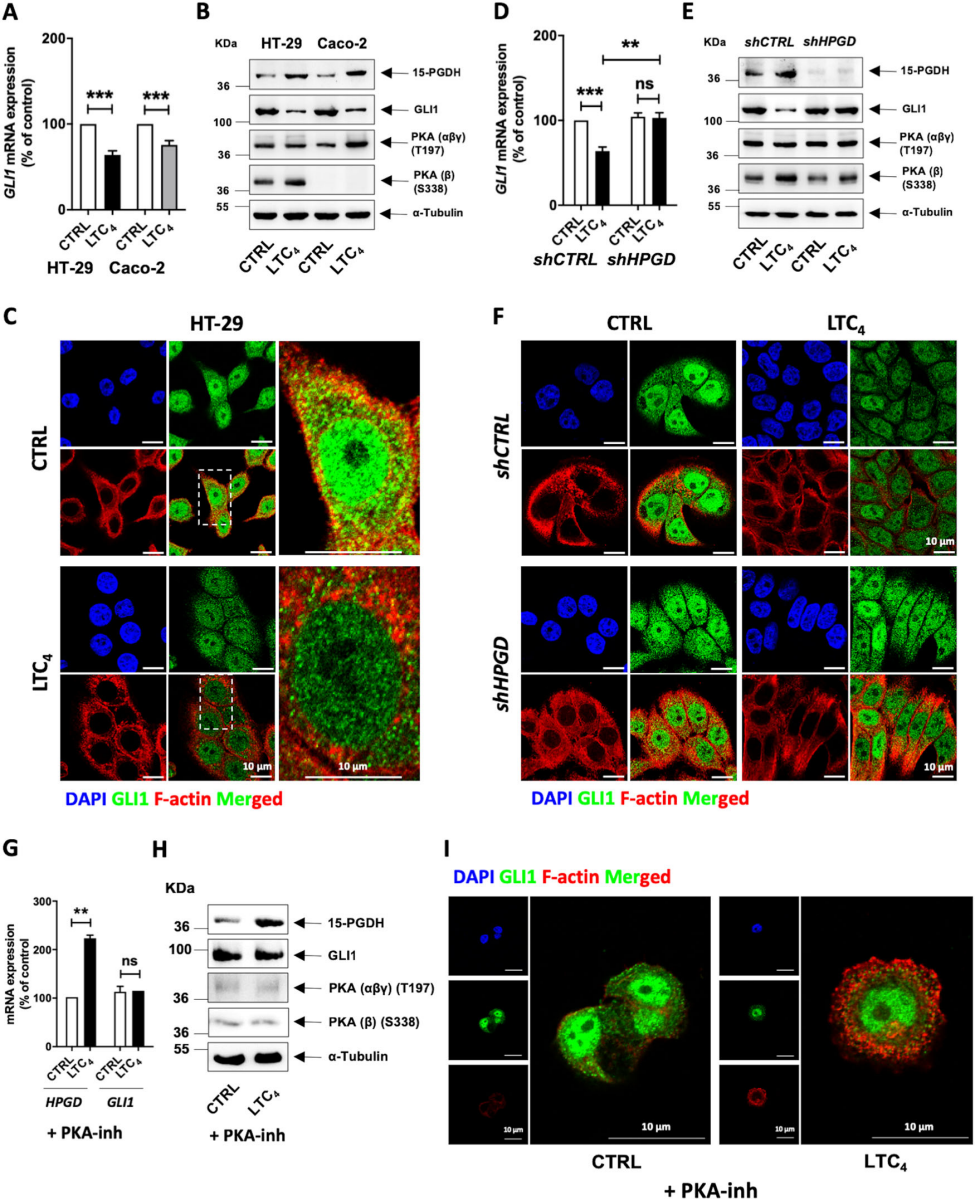
15-PGDH regulates the LTC_4_-mediated downregulation of Hh–GLI signalling in colon cancer cells. **a** qRT-PCR analysis of *GLI1* mRNA expression in HT-29 and Caco-2 cells with or without LTC_4_ stimulation for 48 h. **b** Western blot analysis of 15-PGDH, GLI1, and phospho-PKA (αβγ subunit and β subunit) levels in HT-29 and Caco-2 cells with or without LTC_4_ stimulation. α-Tubulin served as the loading control. **c** Immunofluorescence analysis of GLI1 in HT-29 cells with or without LTC_4_ stimulation for 48 h. **d** qRT-PCR analysis of HT-29 cells transfected with control shRNA (*shCTRL*) or PGDH-specific shRNA (*shHPGD*) with or without LTC_4_ stimulation for 48 h. **e** Western blot analysis of HT-29 cells transfected with control shRNA (*shCTRL*) or PGDH-specific shRNA (*shHPGD*) blotted with antibodies against 15-PGDH, GLI1, or phospho-PKA (αβγ subunit and β subunit) with or without LTC_4_ stimulation for 48 h. α-Tubulin served as the loading control. **f** Immunofluorescence analysis of GLI1 in HT-29 cells transfected with control shRNA (*shCTRL*) or PGDH-specific shRNA (*shHPGD*) with or without LTC_4_ stimulation for 48 h. **g** qRT-PCR analysis of *15-PGDH* and *GLI1* in HT-29 cells treated with the PKA inhibitor H89 (PKA-inh) for 6 h followed by LTC_4_ for 48 h. **h** Western blot analysis showing the expression of 15-PGDH, GLI1, and phospho-PKA (αβγ subunit and β subunit) in HT-29 cells treated with the PKA inhibitor H89 (PKA-inh) for 6 h followed by LTC_4_ for 48 h. **i** Immunofluorescence analysis of GLI1 in HT-29 cells treated with the PKA inhibitor H89 (PKA-inh) for 6 h followed by LTC_4_ for 48 h. *HPRT1* was used as the housekeeping gene for normalisation of the qRT-PCR gene expression data. Graphs represent the mean ± SEM of data from 3 to 4 independent experiments, **P < 0.01, ***P < 0.001.

We determined whether Hh–GLI signalling was involved in the LTC_4_-induced 15-PGDH-promoted differentiation of CC cells^20^. Interestingly, LTC_4_ stimulation significantly downregulated GLI1 expression at both the mRNA and protein levels compared with unstimulated HT-29 and Caco-2 cells (Fig. 3a, b). In addition, immunofluorescence analysis of GLI1 expression revealed a decrease in nuclear GLI1 upon LTC_4_ stimulation (Fig. 3c). To determine the mechanism of 15-PGDH-mediated depletion of GLI1, we examined the expression of protein kinase A (PKA), which is a known GLI1 antagonist^25,26^. The activation of the PKA (α/β/δ) catalytic subunit in Caco-2 cells after stimulation by LTC_4_ was significantly increased, as indicated by the levels of phosphorylated PKA threonine 197, but remained unchanged in stimulated HT-29 cells (Fig. 3b). Moreover, phosphorylation at serine 338 on the catalytic subunit of PKA (β) was increased after LTC_4_ stimulation in HT-29 cells but was not present in Caco-2 whole-cell lysates (Fig. 3b).

15-PGDH-specific shRNA (*shHPGD*) was employed to investigate the involvement of 15-PGDH in the LTC_4_-mediated downregulation of GLI1 expression. Compared with cells transfected with control shRNA (*shCTRL*), CC cells transfected with *shHPGD* showed no response to LTC_4_ stimulation. We observed that shRNA-mediated knockdown of *HPGD* did not affect the expression of the intestinal differentiation markers *CDX2* or *CDHR2* or the Wnt target *AXIN2* (Supplementary Fig. S2A–D for HT-29 cells) and unaltered *GLI1* expression at both the mRNA and protein levels (Fig. 3d–f; see Supplementary Fig. S3A–F for Caco-2 cells). Similarly, 15-PGDH knockdown reduced the effect of LTC_4_ stimulation on PKA activation in both HT-29 and Caco-2 cells (Fig. 3e; Supplementary Fig. S3F).

To further confirm the role of PKA as an intermediate molecule in LTC_4_-induced 15-PGDH-mediated downregulation of GLI1, we used H89, a PKA-specific inhibitor (135 nM), prior to stimulating the cells with LTC_4_. We found that neither HT-29 nor Caco-2 cells treated with the PKA inhibitor affected LTC_4_-induced 15-PGDH expression at either the mRNA or protein levels. However, no significant alteration was observed in either the mRNA or protein level of GLI1 post-stimulation with LTC_4_ (Fig. 3g, h; Supplementary Fig. S4A, B), which was also supported by immunofluorescence analysis (Fig. 3i; Supplementary Fig. S4C). These data indicate that PKA plays a role in the 15-PGDH-mediated downregulation of GLI1 in colon cancer cells.

### GLI1 regulates 15-PGDH-promoted differentiation in colon cancer cells

**Fig. 4:**
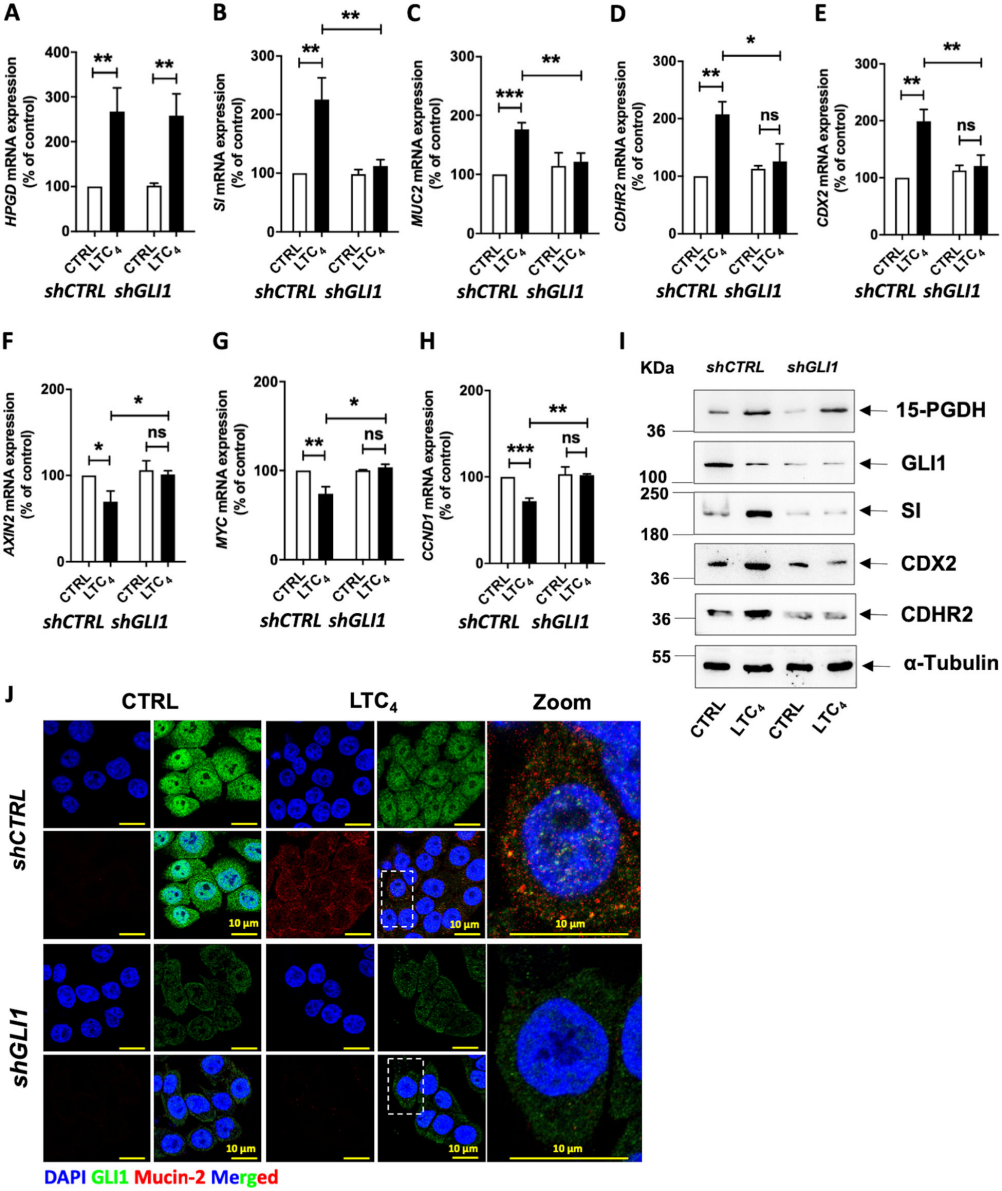
15-PGDH promotes differentiation in colon cancer via GLI1. qRT-PCR validation of gene expression in *shCTRL* (control shRNA)- or *shGLI1* (*GLI1*-specific shRNA)-transfected HT-29 cells with or without LTC_4_ stimulation for 48 h. Marker of tumour suppression in **a** *15-PGDH*. Markers of differentiation in **b** *SI*, in **c** *MUCIN-2*, and in **d** *CDHR2*. Marker of differentiation regulation as in **e** *CDX2*. Markers of Wnt activation in **f** *AXIN2* and **g** *cMYC*. Marker of proliferation in **h** (*CCND1*). *HPRT1* was used as the housekeeping gene for normalization of the gene expression data. **i** Western blot analysis of whole-cell lysates for 15-PGDH, GLI1, SI, CDX2, and CDHR2 expression in *shCTRL* (control shRNA)- or *shGLI1* (*GLI1*-specific shRNA)-transfected HT-29 cells with or without LTC_4_ stimulation for 48 h. α-Tubulin served as the loading control. **j** Immunofluorescence analysis of GLI1 and Mucin-2 in cells with or without LTC_4_ stimulation for 48 h and transfected with *shCTRL* or *shGLI1*. Graphs represent the mean ± SEM of data from three independent experiments, *P < 0.05, **P < 0.01, ***P < 0.001.

Next, we determined the mechanism by which LTC_4_-induced 15-PGDH promoted differentiation in colon cancer cells^20^. Based on the above evidence, we investigated the effects of *GLI1* knockdown (Fig. 4a–j for HT-29 cells and Supplementary Fig. S5A–K for Caco-2 cells) on *HPGD* (15-PGDH) expression. We used *shGLI1* to determine possible changes in the expression of 15-PGDH. However, *GLI1* knockdown in these cells did not affect LTC_4_-induced 15-PGDH expression at either the mRNA or protein level compared with the corresponding *shCTRL*-transfected cells. These data indicate that the effect of LTC_4_ signalling on 15-PGDH expression is independent of GLI1, suggesting that GLI1 is downstream of 15-PGDH. We next validated the involvement of GLI1 in differentiation by testing the mRNA expression of *SI* (sucrase–isomaltase) and *MUC2* (Mucin-2), which are representative intestinal differentiation markers, following LTC_4_ stimulation. The observed increases in the mRNA and protein expression of SI after LTC_4_ stimulation in both HT-29 (Fig. 4b) and Caco-2 cells (Supplementary Fig. S5C) were abolished in *GLI1*-knockdown cells (Fig. 4b; Supplementary Fig. S5C, J). Furthermore, similar results were observed for the mRNA expression of other differentiation markers, such as *MUC2* (Fig. 4c; Supplementary Fig. S5D), and for both the mRNA and protein expression of CDHR2 and CDX2 (Fig. 4d, e; Supplementary Fig. S5E, F, J). LTC_4_ induced a reduction in *AXIN2*, a potential Wnt signalling target (Fig. 4f; Supplementary Fig. S5G). Furthermore, *MYC* and *CCND1* mRNA expression was also abolished in *GLI1*-knockdown cells exposed to LTC_4_ (Fig. 4g, h; Supplementary Fig. S5H, I). To further validate the above observations, we performed western blot (Fig. 4i) and immunofluorescence microscopy using double staining for GLI1 and Mucin-2 in *shCTRL-* and *shGLI1*-transfected cells and found that Mucin-2 expression was downregulated in cells lacking GLI1 (Fig. 4j; Supplementary Fig. S5K). These findings support our hypothesis of a possible regulatory effect of GLI1 in LTC_4_-induced, 15-PGDH-promoted differentiation in colon cancer cells.

### GLI1 suppresses differentiation in the absence of 15-PGDH

**Fig. 5:**
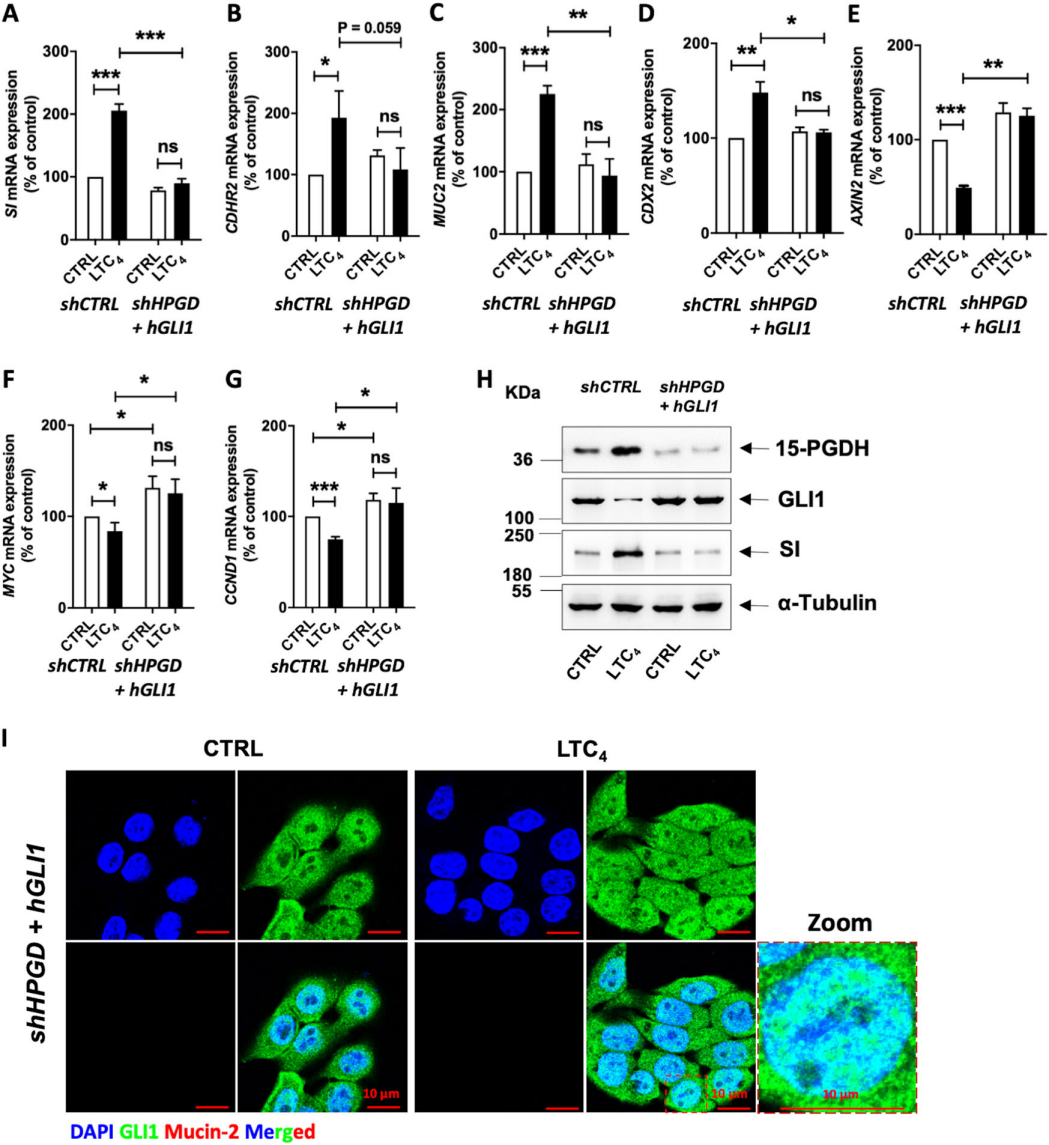
GLI1 negatively regulates the differentiation and promotes the proliferation of colon cancer cells in the absence of 15-PGDH. HT-29 cells were either transfected with *shCTRL* alone or co-transfected with *shHPGD* and *pEGFP-hGLI1* followed by LTC_4_ stimulation for 48 h. qRT-PCR analysis of the differentiation markers **a** *SI*, **b** *MUCIN-2* and **c** *CDHR2*, **d** the differentiation regulation marker *CDX2*, **e** the Wnt activation marker *AXIN2*, **f** the pro-oncogene *cMYC* and **g** the proliferation marker *CCND1*. **h** Western blot analysis of whole-cell lysates for 15-PGDH, GLI1, and SI expression. α-Tubulin was used as the loading control. **i** Immunofluorescence analysis of GLI1 and Mucin-2 in unstimulated or LTC_4_-stimulated cells transfected with *shHPGD* or *shGLI1*. *HPRT1* was used as the housekeeping gene for normalization of the qRT-PCR gene expression data. Graphs represent data from 3 to 4 independent experiments and represent the mean ± SEM, *P < 0.05, **P < 0.01, ***P < 0.001.

We next investigated the ability of GLI1 to regulate 15-PGDH by overexpressing GLI1 using the *pEGFP-hGLI1* construct in combination with the simultaneous knockdown of *HPGD* with *shHPGD* (Supplementary Figs. S6A, B; S7A, B). We found significant downregulation in the LTC_4_-induced increase in *SI*, *MUC2*, *CDHR2*, and *CDX2* mRNA expression levels in HT-29 cells (Fig. 5a–d) and also in Caco-2 cells (Supplementary Fig. S7C–F) that overexpressed *GLI1* and lacked *15-PGDH* compared with cells expressing the control vector (*shCTRL)*. We also found that the LTC_4_-induced reduction in *AXIN2*, *MYC*, and *CCND1* mRNA was abolished, although we observed increased basal levels of these mRNAs (Fig. 5e–g; Supplementary Fig. S7G–I). We next investigated the protein expression of SI by western blot (Fig. 5h; Supplementary Fig. S7J) and Mucin-2 by immunofluorescence microscopy (Fig. 5i; Supplementary Fig. S7K). The LTC_4_-induced increase in SI was abolished in *HPGD*-knockdown and *GLI1-*overexpressing cells (Fig. 5h; Supplementary Fig. S7J). A similar pattern was found regarding Mucin-2 protein expression in these cells, which was observed using immunofluorescence microscopy (Fig. 5i; Supplementary Fig. S7K). Finally, the LTC_4_-induced reduction in GLI1 was abolished (Fig. 5i).

### LTC_4_-induced CysLT_2_R signalling downregulates GLI1 in a 15-PGDH-dependent manner

**Fig. 6:**
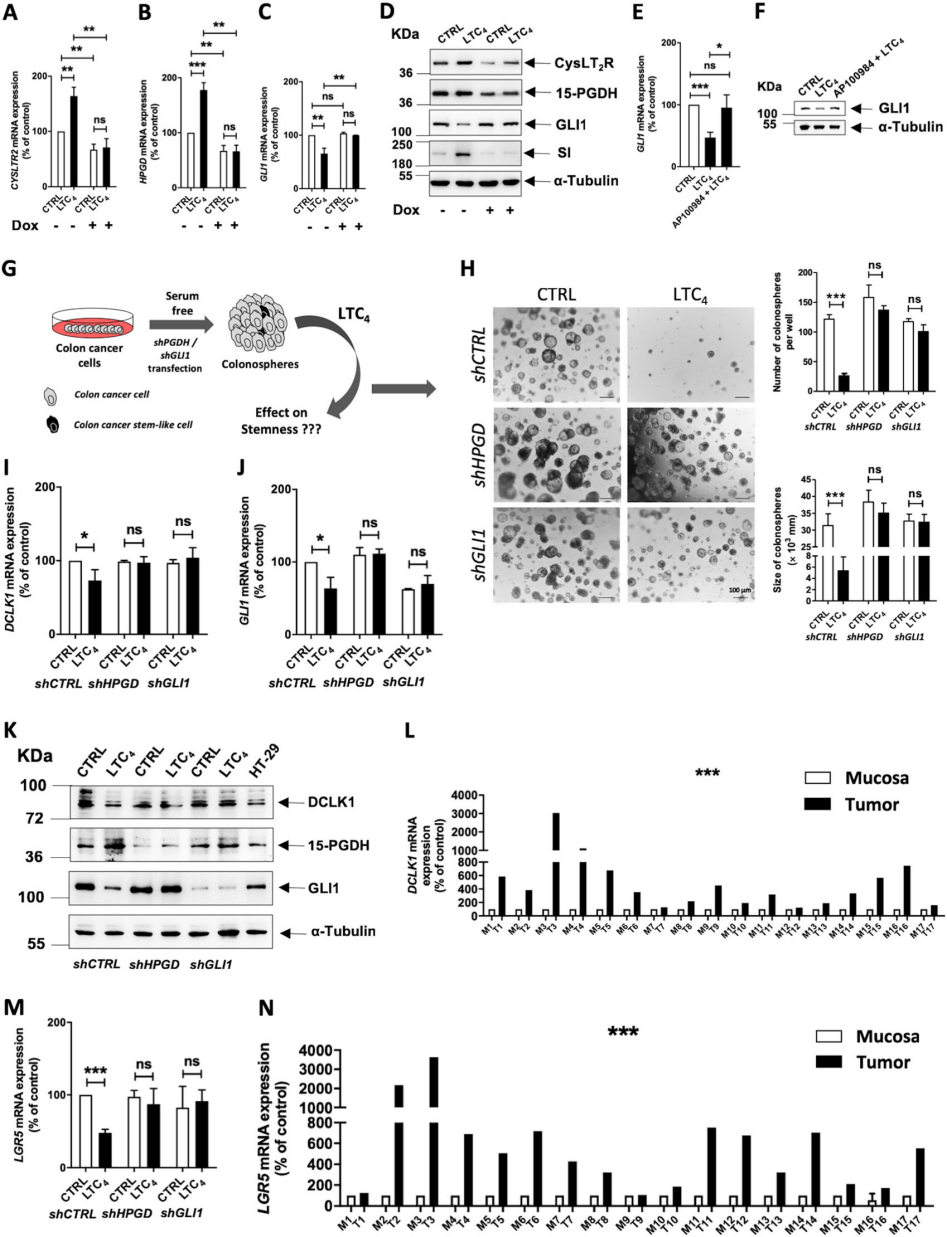
LTC_4_-induced 15-PGDH expression negatively regulates GLI1 via CysLT_2_R. Graphs showing qRT-PCR analysis of **a** *CYSLTR2*, **b** *15-PGDH*, and **c** *GLI1* mRNA expression in HCT-116 cells with stable transfection of doxycycline-regulated *shCYSLTR2* cultured with or without doxycycline (1 µM) treatment followed by LTC_4_ (40 nM) stimulation for 48 h. **d** Western blot analysis of CysLT_2_R, 15-PGDH, GLI1, and SI expression in whole-cell lysates. α-Tubulin served as the loading control. **e** qRT-PCR analysis showing mRNA expression of *GLI1* and **f** western blot analysis of GLI1 protein expression in HT-29 cells stimulated with or without LTC_4_ and with or without AP100984 (a CysLT_2_R antagonist). *HPRT1* was used as the housekeeping gene, and α-tubulin was used as the loading control in the western blot assay. **g** Schematic illustration of colonosphere formation. GLI1 regulates the effect of LTC_4_ on stemness in multicellular colonospheres. The cells were cultured in ultra-low-attachment conditions on matrigel containing serum-free medium for 14 days. **h** Representative images of colonospheres from HT-29 cells transfected with *shCTRL*, *shHPGD*, or *shGLI1* and stimulated or not stimulated with LTC_4_. Bar graphs showing the number of colonospheres formed per well and the size of colonospheres with or without LTC_4_ stimulation and comparing the *shCTRL*-, *shHPGD*-, and *shGLI1*-transfected groups. qRT-PCR analysis of the stemness markers **i** *DCLK1*, **j** *GLI1*, and **m** *LGR5* in colonospheres derived from *shCTRL-*, *shHPGD-*, or *shGLI1*-transfected HT-29 cells with or without LTC_4_ stimulation for 48 h. **k** Western blot analysis showing the expression of DCLK1, 15-PGDH, and GLI1 in transfected HT-29 cells as indicated. α-Tubulin served as the loading control. qRT-PCR analysis of **l** *DCLK1* and **n** *LGR5* in matched pairs of mucosa (M) and tumour (T) tissues from CRC patients (*n* = 17). *HPRT1* was used as the housekeeping gene for normalization of the qRT-PCR gene expression data. Data represent the mean ± SEM from 4 to 5 independent experiments, *P < 0.05, **P < 0.01, ***P < 0.001.

To determine whether LTC_4_ acts via CysLT_2_R, as LTC_4_ is the high-affinity ligand of CysLT_2_R, we investigated the specific involvement of CysLT_2_R signalling in the LTC_4_-mediated downregulation of GLI1 in colon cancer cells. We first constructed HCT-116 cells with doxycycline (Dox)-inducible stable knockdown of CysLT_2_R (*shCYSLTR2*)^27^. We examined the mRNA and protein expression levels of CysLT_2_R, 15-PGDH, and GLI1 with and without LTC_4_ stimulation in this cell line with Dox induction and compared them with the levels in cells grown in the absence of Dox. The mRNA expression of *CYSLTR2* and *HPGD* was significantly upregulated (Fig. 6a, b), while *GLI1* mRNA expression was downregulated in cells cultured without Dox (Fig. 6c). However, in the Dox-induced cells, *GLI1* gene expression remained unaltered, most likely due to the low mRNA expression levels of *CYSLTR2* and *HPGD*. These observations were also reflected in the levels of protein expression (Fig. 6d; Supplementary Fig. S8A–C).

To further study the involvement of CysLT_2_R, we treated HT-29 and Caco-2 cells with the CysLT_2_R-specific antagonist AP100984 (1 µM) followed by LTC_4_ stimulation for 48 h^20^. AP100984 treatment efficiently blocked both the mRNA (Supplementary Fig. S8D, E) and protein expression (Supplementary Fig. S8F) of CysLT_2_R as well as of its downstream signal 15-PGDH in HT-29 cells as well as in Caco-2 cells (Supplementary Fig. S8G–J). AP100984 pre-treatment abolished the effect of LTC_4_ stimulation on GLI1 at both the mRNA and protein levels (Fig. 6e, f; Supplementary Fig. S8I, J). Taken together, the above results showed that CysLT_2_R signalling plays a role in the LTC_4_-induced 15-PGDH-mediated downregulation of GLI1.

### LTC_4_-induced 15-PGDH expression reduces stemness in colonospheres

We extended our study to determine whether LTC_4_-induced 15-PGDH affected stemness in colon cancer cells as well as the possible role of GLI1. We created a 3D model of multicellular colonospheres derived from colon cancer cells (Fig. 6g). We observed that *shCTRL*-transfected HT-29 or Caco-2 cell-derived colonospheres showed reduced numbers and sizes with LTC_4_ stimulation (Fig. 6). Unlike the *shCTRL* group, *shHPGD*-transfected cell-derived colonospheres showed increases in number and size. However, the absence of GLI1 in *shGLI1*-transfected cell-derived colonospheres resulted in no significant alteration in size or number even after LTC_4_ stimulation. The mRNA expression levels of the colon cancer-specific stemness markers *DCLK1*, *LGR5*, and *ALDH1A1* were elevated in HT-29 as well as in Caco-2 cell-derived colonospheres and were downregulated after LTC_4_ stimulation (Fig. 6i; Supplementary Fig. S9A–H). The mRNA and protein levels of the cancer stem cell markers DCLK1 and ALDH1A1 remained unchanged in both *shHPGD*- and *shGLI1*-knockdown HT-29 and Caco-2 cell-derived colonospheres, even after LTC_4_ stimulation, compared with their unstimulated counterparts (Fig. 6i–k; Supplementary Fig. S9A–H). GLI1 gene and protein expression in HT-29- and Caco-2-derived colonospheres was also downregulated after LTC_4_ stimulation. We next analysed the mRNA expression levels in tumour and adjacent mucosa samples from 17 paired CRC patients, which also suggested a significantly positive correlation between *GLI1* and the stemness markers *DCLK1* and *LGR5* with elevated expression of all three genes in tumour tissues (Figs. 1l, 6l–n). The normal mucosa as reference, set to 100 and the tumour tissue for *DCLK1* 563.11 ± 166.84 and *LGR5* 722.40 ± 215.94 (mean ± SEM) respectively, Mann–Whitney test, *P* < 0.001. As expected, the expression level of *DCLK1* showed a negative correlation with *CYSLTR2* and *HPGD* expression from our previously published results (see ref. ^20^), while *MUC2* had a positive correlation (Fig. 2c)^20^. This suggests that a poorly differentiated tumour could occur due to enriched stemness.

### LTC_4_-induced 15-PGDH promotes differentiation in zebrafish xenografts

**Fig. 7:**
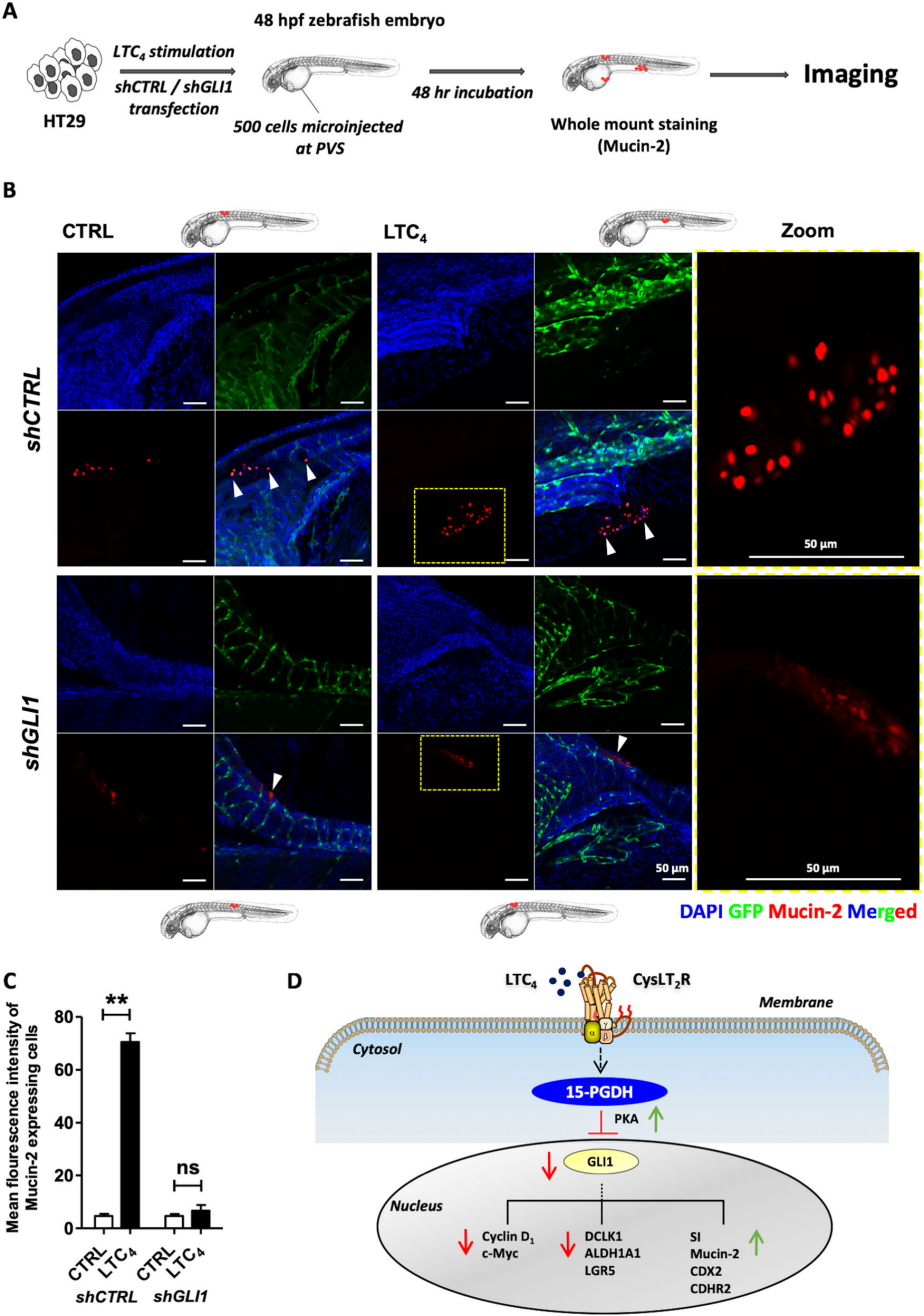
LTC_4_-induced 15-PGDH promotes differentiation in transgenic zebrafish xenografts. **a** Schematic diagram showing the experimental setup of HT-29 cell-based xenografts in transgenic zebrafish (*n* = 20 in each group). **b** Immunofluorescence analysis of whole-mount staining of xenografted transgenic zebrafish *Tg*(*fli1:EGFP*) embryos with anti-Mucin-2 antibodies after the injection of *shCTRL-* or *shGLI1*-transfected HT-29 cells with or without LTC_4_ stimulation into the perivitelline space (PVS)^53^. **c** Graphical representation of the mean fluorescent intensity of Mucin-2-expressing HT-29 colon cancer cells transfected with *shCTRL* or *shGLI1* with or without LTC_4_ stimulation. The scale bars are as indicated in the images. **d** Schematic mechanistic model described in the study. Mean ± SEM, **P < 0.01.

Next, we used the zebrafish xenograft model^28^ to further evaluate and visualise the differentiation-promoting role of 15-PGDH in colon cancer. Transgenic zebrafish *Tg*(*fli1:EGFP*) embryos were injected with unstimulated or LTC_4_-stimulated HT-29 cells and incubated for 48 h (Fig. 7a). We found a twofold increase in Mucin-2 expression in zebrafish xenografts injected with LTC_4_-stimulated HT-29 cells compared with zebrafish xenografts injected with unstimulated cells (Fig. 7b). Furthermore, Mucin-2 expression was downregulated in zebrafish embryos injected with *shGLI1*-transfected HT-29 cells even after LTC_4_ stimulation (Fig. 7c). This observation further strengthened the conclusion that GLI1 is involved in regulating the redifferentiation of CC cells.

## Discussion

Previously, we reported an antitumour effect of LTC_4_ via the induction of 15-PGDH. In this study, we focused on the role of GLI1 in the context of LTC_4_/CysLT_2_R signalling in colon cancer. In addition to hyperactivated Wnt/β-catenin signalling, canonical Hh signalling acts as a prominent regulator of colonic tumorigenesis^4,7^. Canonical Hh signalling involves the coordinated action of the patch family transmembrane receptor PTCH, the intermediate molecule Smoothened (SMO) and GLI, which is a transcriptional regulator downstream of sonic hedgehog (SHH). Under normal physiological conditions, Hh signalling regulates embryogenesis, development, and differentiation^29^; lon carcinoma^4,7^. Although GLI1 has been targeted in other solid tumours^30–32^, it has never been addressed whether Hh–GLI inhibition could promote differentiation.

In this study, the promotion of differentiation in colon cancer cells was found to occur via the inhibition of canonical Hh/GLI1 signalling. We demonstrated that the induction of 15-PGDH by LTC_4_/CysLT_2_R signalling in cells downregulated GLI1, a prominent downstream target of Hh signalling. LTC_4_ induced significant phosphorylation of the subunits β (S338) and/or αβγ (T197) of PKA, a well-known GLI1 antagonist, in HT-29 and Caco-2 cells. The involvement of PKA was further confirmed by using a specific inhibitor (H89). Although these cells exclusively engage different subunits of PKA, these findings provide substantial evidence that PKA is an intermediate player involved in the 15-PGDH-mediated decrease in GLI1 expression. The decrease in GLI1 expression led to the redifferentiation and reduction in the proliferation marker *CYCLIND1* in cells. Furthermore, in multicellular colonospheres, LTC_4_-CysLT_2_R signalling decreased the expression of the stemness markers *ALDH1A1*, *DCLK1*, and *LGR5* in a 15-PGDH/GLI1-dependent manner, which supported the conclusions of a previous study^4^ in which Varnat et al. showed the exclusive involvement of Hh–GLI1 signalling in the enrichment of CC stemness. Moreover, we used an AOM/DSS-induced CAC model and validated the involvement of CysLT_2_R signalling in coordinating this process. In cells obtained from mouse intestine and colon cancer patients, we identified GLI1 as a downstream regulator of 15-PGDH-induced redifferentiation. Transgenic zebrafish xenografted with HT-29 cells revealed a GLI1-dependent increase in Mucin-2-expressing cells after LTC_4_ stimulation. When we compared the expression levels of *GLI1* and *DCLK1* or *LGR5* between matched pairs of normal and cancer tissues from CRC patients, we found that their expression levels were negatively correlated with *MUCIN-2* expression in cancer tissue.

The PGE_2_-degrading enzyme 15-PGDH has been recently studied to determine its antitumour potential in solid tumours such as pancreas and lung cancer^33,34^ in addition to colon cancer^19,20,35^. Some studies have focused on the antitumour role of 15-PGDH through PGE_2_ degradation^36,37^, whereas the findings of other studies have implied a differentiation-promoting role^20^. Two consecutive studies by Kangwan et al. showed the antitumour role of SHH inhibitors against colonic carcinogenesis, which was regulated through an IL-6/STAT-3 axis that was also involved in 15-PGDH activation^38,39^. These findings are in contrast with those found in the present study, where we found that 15-PGDH activation inhibited Hh signalling.

Previous studies by our group have revealed the antitumour role of CysLT_2_R signalling via promotion of the redifferentiation of colon cancer cells with the activation of alkaline phosphatase and Mucin-2 expression after LTC_4_ and IFN-α stimulation^12^. Furthermore, Bengtsson et al. emphasized the role of all-trans retinoic acid in activating CysLT_2_R and promoting differentiation in colon cancer cells^40^. An additional study by Magnusson et al. clearly demonstrated the correlation between high CysLT_2_R expression and both higher differentiation levels in colon cancer patients and LTC_4_-induced differentiation markers in colon cancer cells^41^. These previous studies support our current observations that LTC_4_-mediated CysLT_2_R activation promoted a redifferentiation state, which seems to be regulated by GLI1. Recently, Mehdawi et al. reported that the activation of LTC_4_-induced CysLT_2_R signalling led to the activation of 15-PGDH, which further promoted the differentiation of colon cancer cells in a 15-PGDH-dependent manner, as evidenced by the upregulation of intestinal differentiation markers^20^. The current study determined a hitherto unknown underlying regulatory mechanism of the LTC_4_-induced 15-PGDH-mediated promotion of redifferentiation in colon cancer cells.

It is reasonable to speculate that a decrease in proliferation and stemness would be observed in cells undergoing differentiation. Moreover, Wnt signalling is known to contribute greatly to proliferation and stemness in CRC cells. Based on this background, we hypothesised that LTC_4_-mediated 15-PGDH induction may also have a negative effect on Wnt target proteins. We therefore investigated the mRNA expression of the Wnt target genes *AXIN2*, *cMYC*, and *CYCLIND1*, which are closely associated with cellular proliferation and stemness enrichment. LTC_4_-induced 15-PGDH downregulated Wnt target genes in a *GLI1*-dependent manner with concurrent activation of the intestinal tumour suppressor *CDX2*, which positively regulates the expression of SI in colon cancer cells^24,42^ and is a prominent intestinal differentiation marker. In adult intestines, *CDX2* controls the balance between proliferation and differentiation, and its depletion results in dedifferentiation, leading to cancer pathogenesis^43,44^. In CRC, *CDX2* is highly suppressed^45^ and is inversely correlated with disease progression and metastasis^46,47^. However, previous reports suggested that activated *CDX2* regulates *AXIN2* expression in Caco-2 cells^42,48^. Moreover, it regulates the Wnt degradation complex consisting of AXIN2, APC/GSK-3β, and CK-1, and it re-establishes a differentiation state^49^. The relationship between *CDX2* and *CYCLIND1* and the *cMYC* oncogene has been elucidated previously^50,51^. Taken together, our observations support the positive correlation between *CDX2* and *SI* as well as the negative regulatory action of *CDX2* on the designated Wnt targets *AXIN2*, *cMYC*, and *CYCLIND1* in colon cancer cells.

Our in vivo model results suggest a negative correlation between GLI1 expression and CysLT_2_R signalling. Moreover, IHC analysis of CRC patient tissues showed a significant negative correlation between GLI1, 15-PGDH and Mucin-2 expression in CRC tissue, which is in line with the mRNA data from the public CRC database. Although we did not observe a significant correlation between GLI1 and CysLT_2_R expression in our patient cohort, the public data from Sveen et al. show a weakly negative but significant correlation between the mRNA expression levels of *GLI1* and *CYSLTR2*, which is further supported by our in vitro and in vivo findings. Furthermore, the mRNA analysis of paired normal and cancer tissues from patients suggested that *GLI1* and stemness were positively correlated (Figs. 1l, 6l, n) and coupled with adverse effects on differentiation-promoting factors (Fig. 2c). The protein expression of CysLT_2_R and 15-PGDH in matched normal vs tumour tissue showed a negative correlation with GLI1 expression (Fig. 1k). Therefore, the LTC_4_/CysLT_2_R/15-PGDH signalling-mediated downregulation of Hh–GLI1 signalling combined with increased redifferentiation could be an alternative therapeutic treatment in addition to classical chemotherapy for colon cancer patients (Fig. 7d).

According to this concept, we speculate that inducing 15-PGDH signalling via CysLT_2_R activation in CRC patients might lead to new treatment opportunities^52^ and better clinical outcomes by downregulating GLI1 and hence guiding the cancer cells into a more redifferentiated state.

## Materials and methods

### Antibodies and reagents

Please see Supplementary Materials and Methods.

### Animal care and handling

Please see Supplementary Materials and Methods.

### Colonosphere formation and quantification

Please see Supplementary Materials and Methods.

### Dox-inducible CysLT2R stable knockout cell line

Please see Supplementary Materials and Methods.

### Cell lines and reagents

Please see Supplementary Materials and Methods.

### Western blot analysis

Please see Supplementary Materials and Methods.

### Immunofluorescence analysis

Please see Supplementary Materials and Methods.

### Immunohistochemistry

Please see Supplementary Materials and Methods.

### Patients

Please see Supplementary Materials and Methods.

### Ethical statement

Please see Supplementary Materials and Methods.

### Public database

Please see Supplementary Materials and Methods.

### Quantitative real-time PCR

Please see Supplementary Materials and Methods.

### Short-hairpin RNA (shRNA) and plasmid transfection

Please see Supplementary Materials and Methods.

### Zebrafish xenografts

Please see Supplementary Materials and Methods.

### Statistical analysis

Please see Supplementary Materials and Methods.

## Supplementary information

Supplementary Materials and Methods

Supplementary Figure S1

Supplementary Figure S2

Supplementary Figure S3

Supplementary Figure S4

Supplementary Figure S5

Supplementary Figure S6

Supplementary Figure S7

Supplementary Figure S8

Supplementary Figure S9
