# Supplementary material for: Tumour suppressor 15-hydroxyprostaglandin dehydrogenase induces differentiation in colon cancer via GLI1 inhibition
Source: Oncogenesis. 2020 Aug 19;9(8):74. doi: 10.1038/s41389-020-00256-0 (PMC7438320; doi:10.1038/s41389-020-00256-0)
Supplement: Supplementary file 1 — Supplementary Materials and Methods [file 41389_2020_256_MOESM1_ESM.docx]

**Supplementary Materials and Methods**

# Animal care and handling

We used a colitis-associated colon cancer (CAC) model of wild-type and *cysltr2^-/-^* mice on a C57BL/6N background in accordance with a previously described protocol ^24^. Transgenic zebrafish (*Danio rerio*), *Tg*(*fli1:EGFP*), were handled and used in accordance with the national legislation of Sweden and the European Community guidelines for animal studies. All procedures were approved by the Ethical Committee at Malmö-Lund (permit no. M23-15, zebrafish larvae) ^28^.

# Antibodies and reagents

The primary antibodies anti-CysLT_2_R (SC-27096), anti-Mucin-2 (SC-23171), anti-DCLK1 (SC-46312), and anti-α-tubulin (SC-32293) were procured from Santa Cruz Biotechnology, Dallas, TX, USA and used for immunoblotting and immunofluorescence, as described herein. Anti-PKA (α/β/γ catalytic subunit, phospho-T197; ab75991), anti-PKA (β catalytic subunit, phospho-S338; ab5816), and anti-GLI1 (ab49314) were obtained from Abcam, Cambridge, UK for the immunoblotting and immunofluorescence assays. The PKA inhibitor (H89 dihydrochloride, kinase inhibitor, ab120341) used for cell culture experiments was from Abcam. Anti-GLI1 (ab151796, Abcam), anti-Mucin-2 (SC-15338), anti-CysLT_2_R (C- terminal, Innovagen, Lund, Sweden) and anti-15-PGDH (NB200-179, Novus Biologicals, Centennial, CO, USA) were used for immunohistochemical analysis. Anti-ALDH1A1 (611195, BD Bioscience, USA), anti-SI (SAB2102141, Sigma, St. Louis, MO, USA), anti- CDX2 (3977S, Cell Signaling Technology, Leiden, The Netherlands), and anti-CDHR2 (PCLKC monoclonal antibody (M01) clone 1D2, H00054825-M01, Abnova, Taipei, Taiwan) antibodies were obtained from the specified manufacturers unless otherwise stated. The CysLT_2_R antagonist AP100984 was a kind gift from Dr. J. Evans, Amira Pharmaceuticals (San Diego, CA, USA).

# Cell lines and reagents

Three colon adenocarcinoma cell lines, HT-29 (ATCC^®^ HTB-38™), Caco-2 (ATCC^®^ HTB-37™), and HCT116 (ATCC^®^ CCL-247™), were used in this study. HT-29, HCT116, and HCT116 (Dox-inducible *shCYSLTR2*) cells were cultured in McCoy’s 5A medium (HyClone^™^, GE Healthcare Life Sciences, Pittsburgh, PA, USA) supplemented with L-glutamine (1%), 10% fetal bovine serum, and 100 µg/mL penicillin-streptomycin. Caco-2 cells were maintained in minimum essential medium (MEM; Sigma Life Science, St. Louis, MO, USA) supplemented with L-glutamine (1%), 20% fetal bovine serum, nonessential amino acids (1%), and 100 µg/mL penicillin-streptomycin. HEK293T (ATCC® CRL-11268™) cells were cultured in DMEM (HyClone^™^, GE Healthcare Life Sciences, Pittsburgh, PA, USA) supplemented with L-glutamine (1%), 10% fetal bovine serum, and 100 µg/mL penicillin-streptomycin. All the cell lines were incubated in a humidified incubator with 5% CO_2_ at 37°C. The cells were regularly monitored for mycoplasma infection. The authenticity of the cell lines was confirmed by STR profiling (LGC Standards, cell line authentication service, Teddington, UK).

# Colonosphere formation and quantification

Colonospheres were formed from HT-29 and Caco-2 cells using a previously described protocol ^53^. The number of colonospheres was quantified, and colonosphere size was calculated by measuring the diameter of the spheres with ImageJ software (NIH, Bethesda, MD, USA).

# Dox-inducible CysLT_2_R stable knockout cell line

A cell line with Dox-inducible stable knockdown of CysLT_2_R was established from HCT116 colon cancer cells by following a previously described protocol ^27^. Briefly, HEK293T cells were transfected by the CaPO_4_ method with the pLKO.1 plasmid containing shRNA against *CYSLTR2* and a puromycin control gene in addition to the helper plasmids pHR 8.92 and 8.93 and PMDG2 (VSV envelope protein). The supernatant containing lentiviral particles was harvested after transfection, and the viral particles were used to infect HCT116 cells for 48 h. Viability was monitored post-infection. The GFP-positive populations of HCT116 cells were sorted using a flow cytometry-based sorter (FACS ARIA, BD Biosciences, San Jose, CA, USA; data not shown), and the efficiency of the knockdown of the desired gene was determined by treatment with 1 µM doxycycline (Dox) for 24-72 h. Prior to LTC_4_ stimulation, the cells were cultured in the presence or absence of 1 µM Dox for 48 h.

# Western blot analysis

Proteins were isolated from whole-cell lysates and from the patient tumour and matched normal tissue as described previously ^20^ and analysed by sodium dodecyl sulfate- polyacrylamide gel electrophoresis (SDS-PAGE) on 10% acrylamide gels. The separated proteins were then transferred to a polyvinylidene difluoride (PVDF) membrane and subjected to immunoblotting analysis according to the method described in a previous study^20^.

# Immunofluorescence analysis

Immunofluorescence analysis was performed as previously described ^20^ with some modifications. The cells were cultured and transfected on 12-mm^2^ glass cover slips. After the transfection period, the cells were stimulated with 40 nM LTC_4_ for 48 h. The cells on the

cover slips were fixed with 4% paraformaldehyde at room temperature for 10 min, washed twice with washing buffer (0.1% Tween-20 in phosphate-buffered saline [PBS]), permeabilized with 0.05% Triton-X 100/PBS for 15 min, and then washed twice with washing buffer. The cells were then blocked using 5% bovine serum albumin (BSA)/0.05% PBS-Tween-20 for 1 h at room temperature and incubated overnight at 4 °C with primary antibodies targeting GLI1 (1:500) and Mucin-2 (1:750). The cells were then washed three times with washing buffer and incubated with a secondary antibody (Alexa Fluor-conjugated anti-rabbit or anti-goat, 1:1000 dilution) for 1 h at room temperature. Both the primary and secondary antibodies were diluted in blocking buffer. After incubation with the secondary antibodies, the cells were washed three times in washing buffer, incubated with phalloidin-Alexa Fluor-546-conjugated antibodies for 1 h at room temperature, and then washed once with washing buffer and stained with 4ʹ,6-diamidino-2-phenylindole (DAPI) for 10 min at room temperature. Finally, the cells were washed twice in washing buffer, and the coverslips were mounted on glass slides using Fluoromount G mounting solution (Southern Biotech, Birmingham, AL, USA). Fluorescence images were captured using a Zeiss LSM 700 (Carl Zeiss Microscopy GmbH, Jena, Germany) confocal microscope with a 63× oil objective lens. To obtain a uniform comparison, each group of images was captured using the same laser power and detector gain settings. The images were analysed and processed using LSM Zen (blue edition) software.

# Immunohistochemistry

Immunohistochemical analysis was performed according to a previous report ^22^. After the samples were stained with primary antibodies against GLI1 (1:1000 or 1:250), Mucin-2 (1:100), CysLT_2_R (1:50, overnight) or 15-PGDH (1:200), all the slides were counterstained with haematoxylin and eosin (H&E) and scanned using the Aperio Scanscope XT system (Vista, CA, USA). Two independent investigators (GT and SRS) blindly from patient´s data or mice genotype, assessed the immunoreactivity of Mucin-2 and GLI1 and scored the slides on a scale from 0 to 3 based on the staining intensity (SI) as follows: 0 = negative; 1 = weak; 2 = moderate; and 3 = strong. The percentage of positive cells (PP) was scored as 1, 2, or 3 if the positively stained area was <10%, 11-50%, or >50%, respectively. The IRS was calculated according to the following equation: IRS = SI × PP, where the IRS ranged from 0-9. Patients with missing or incomplete cores were excluded from the analysis.

# Patients

All the patients involved in this investigation belonged to a previously described cohort ^22^. Patients operated for colorectal cancer during 2008-2012 were randomly chosen from four different hospitals in the southern part of Sweden, and the study had 80% sufficient power as calculated and reported in detail´s previously ^22^. The endpoint was overall survival (OS). The follow-up time started at the date of diagnosis and ended at the date of the indicated event (death due to any cause) or censoring (31 August 2016) ^22^.

# Ethical statement

All participating patients gave their written informed consent, and sample collection was made with the approval of the regional research ethics board of Lund University, Sweden (Dnr 3/2006). The studies were per-formed in compliance with the 1975 Declaration of Helsinki, as revised in 1983. The mouse model is approved by the regional ethical committee for animal research at Lund University, who approved the animal experiments (M-263-12).

# Public database

To validate the correlations between GLI1 and CysLT_2_R, 15-PGDH or Mucin-2 analyzed by IHC in patient tissues, public mRNA data from a CRC cohort comprising 333 patients were included in the current study ^23^. The analysis was performed using the electronic database ‘R2: Genomics Analysis and Visualization Platform’ (hgserver1.amc.nl).

# Quantitative real-time PCR

Quantitative real-time polymerase chain reaction (RT-PCR) was performed according to the protocol described in a previous study ^20^. Primers for the following genes were used: *GLI1* (Hs00171790_m1)*, SI* (Sucrase-isomaltase, Hs00356112_m1)*, MUC2* (Mucin-2, Hs00159374_m1), *CDHR2* (Protocadherin-24, Hs00263709_m1)*, HPGD* (15-PGDH*,*

Hs00168359_m1), *AXIN2* (AXIS inhibition protein 2, Hs00610344_m1), *CYSLTR2* (Hs00252658_s1), *ALDH1A1* (Aldehyde dehydrogenase 1 A1, Hs00946916_m1), *DCLK1* (Double cortin-like kinase 1, Hs00178027_m1), *LGR5* (Leucine-rich repeat-containing G-protein coupled receptor 5, Hs00969422_m1), *CDX2* (Caudal type homeobox 2, Hs01078080_m1), *MYC* (*cMYC*, Hs00153408_m1), *CCND1* (Cyclin D1, Hs00765553_m1), and *HPRT1* (Hs99999909_m1). The reactions were analysed using MxPro software on the Mx3005P system (Agilent Technologies, Inc., CA, USA) and normalized against the housekeeping gene *HPRT1.*

# Short-hairpin RNA (shRNA) and plasmid transfection

Short-hairpin RNA (shRNA) and plasmid transfections of the desired genes were performed as described for siRNA transfection in a previous report ^20^. shRNA molecules targeting *PGDH* (sc-61330-SH) and *GLI1* (sc-37911-SH) and the control siRNA-A (sc-37007), siRNA-B (sc-44230), and siRNA-C (sc-44231) were procured from Santa Cruz Biotechnology (Dallas, TX, USA). The *pEGFP-hGLI1* construct was a kind gift from Prof. Rune Toftgård at the Karolinska Institute, Stockholm, Sweden ^51^.

# Zebrafish xenografts

Unstimulated and 40 nM LTC_4_-stimulated HT-29 cells were injected into the perivitelline space (PVS) of anaesthetized zebrafish (*Tg*(*fli1:EGFP*) embryos at 48 h post-fertilization (hpf)^27^. After injection, the embryos were incubated at 32°C until the end of the incubation period and then processed for immunofluorescence-based whole-mount staining.

# Whole-mount staining of zebrafish embryos

Immunofluorescence-based whole-mount staining of zebrafish embryos was performed following a protocol described elsewhere ^52^.

# Statistical analysis

Statistical analysis was performed using GraphPad Prism Version 7.0a (GraphPad Software, Inc., San Diego, CA, USA). The mean values between groups were compared using the

unpaired Student’s *t*-test, Chi-square test and Mann-Whitney U-test as indicated. For the patient results, the SPSS statistical program version 23.0 was used (SPSS, IBM, Armonk, NY, USA). The correlations between GLI1 and CysLT_2_R, 15-PGDH or Mucin-2 IRS were assessed using the Pearson correlation coefficient, and 95% confidence intervals (CIs) were reported. OS curves adjusted for age and TNM stage were generated using the Kaplan-Meier method and compared using the log-rank test. The Cox regression model was used to determine the risk of death between patients with high GLI1 expression and those with low GLI1 expression, and the HR with 95% CI was presented. A P-value < 0.05 was considered statistically significant and all the tests performed were two sided.
