# Supplementary material for: Tumour suppressor 15-hydroxyprostaglandin dehydrogenase induces differentiation in colon cancer via GLI1 inhibition
Source: Oncogenesis. 2020 Aug 19;9(8):74. doi: 10.1038/s41389-020-00256-0 (PMC7438320; doi:10.1038/s41389-020-00256-0)
Supplement: Supplementary file 2 — Supplementary Figure S1 [file 41389_2020_256_MOESM2_ESM.pdf]

Supplementary Fig. S1

A

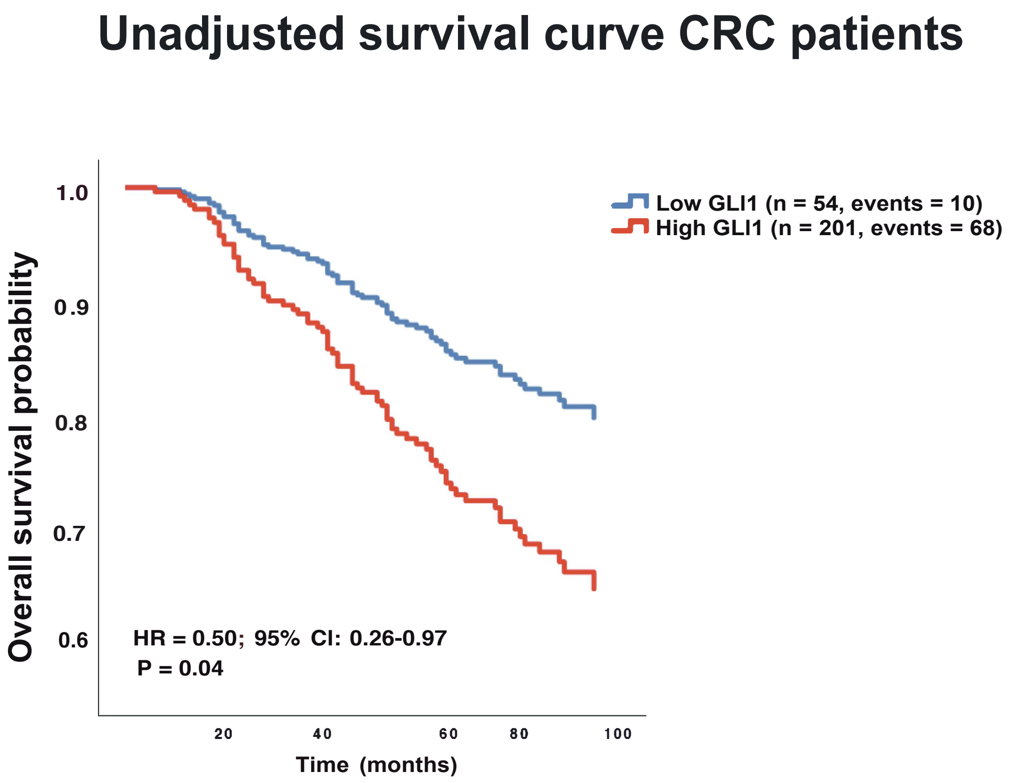

Supplementary Fig. S1A. Unadjusted Kaplan-Meier curves for overall survival of colorectal cancer patients with low and high GLI1 expression for Fig. 1H.

B

Chi-square, df	5.808, 1
z	2.410
P value	0.0159
P value summary	*

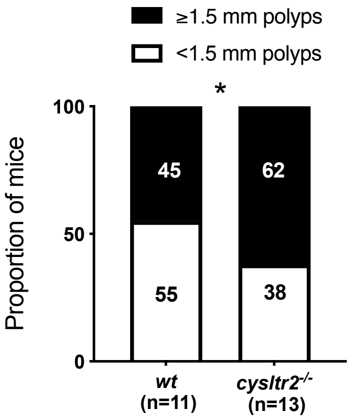

C

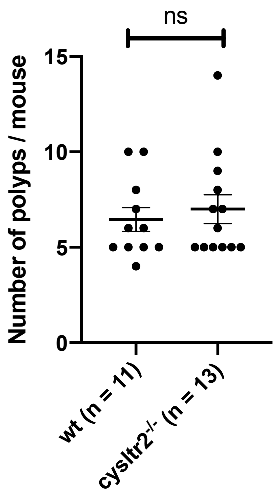

D

wt ***cysltr2$^{-/-}$***

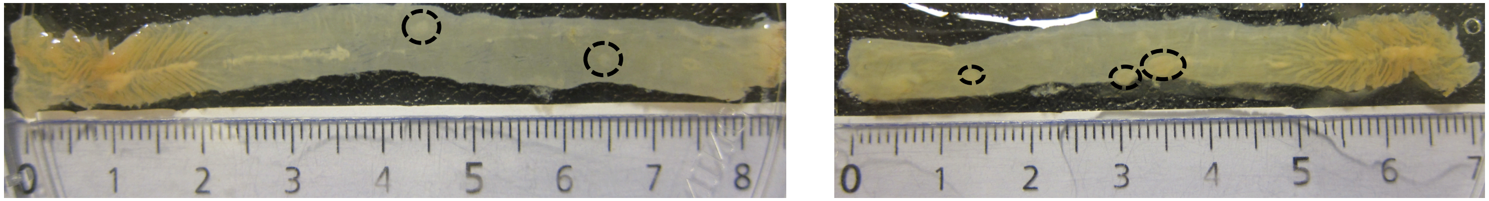

E

10x

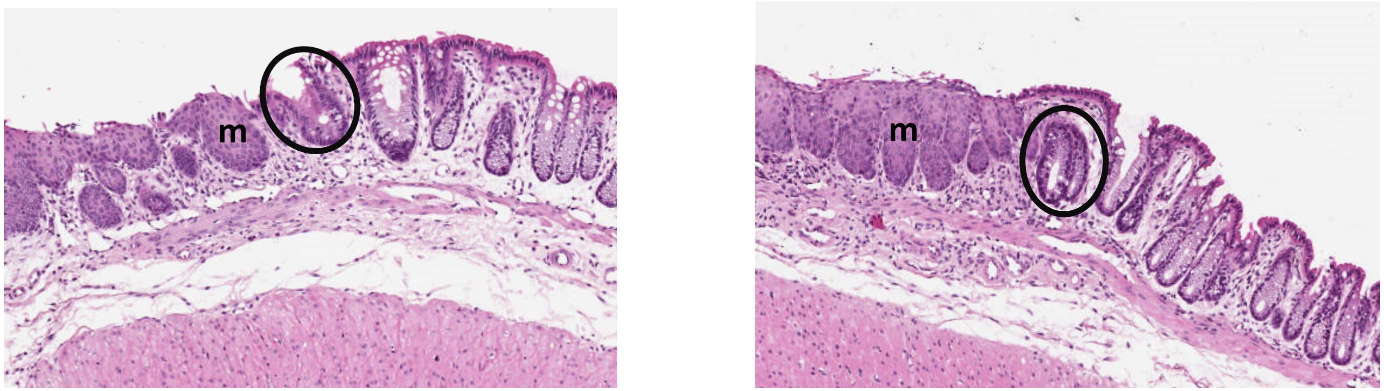

Supplementary Fig. S1B-E.

B. Number of mice with colon polyps < 1.5 mm or ≥ 1.5 mm polyp in AOM/DSS-treated *wt* (n = 11) and *cysltr2*$^{-/-}$ mice (n = 13). The bar diagram outlines the percentage distribution of small and largeer polyps of the respective phenotypes. **C**. The number of polyps, irrespective of size, detected per mouse in AOM/DSS-treated *wt* (n = 11) or *cysltr2*$^{-/-}$ (n = 13) mice. **D.** Representative images from colon of AOM/DSS-treated *wt* or *cysltr2*$^{-/-}$ mice with polyps indicated with dotted circles. **E.** Representative Haemotoxylin and Eosin stained images of colon tissue showing a dysplastic/aberrant crypt focus (black circle) and metaplastic areas (m) from AOM/DSS-treated *wt* and *cysltr2*$^{-/-}$ mice.
