# Supplementary material for: Tumour suppressor 15-hydroxyprostaglandin dehydrogenase induces differentiation in colon cancer via GLI1 inhibition
Source: Oncogenesis. 2020 Aug 19;9(8):74. doi: 10.1038/s41389-020-00256-0 (PMC7438320; doi:10.1038/s41389-020-00256-0)
Supplement: Supplementary file 3 — Supplementary Figure S2 [file 41389_2020_256_MOESM3_ESM.pdf]

Supplementary Fig. S2

HT-29

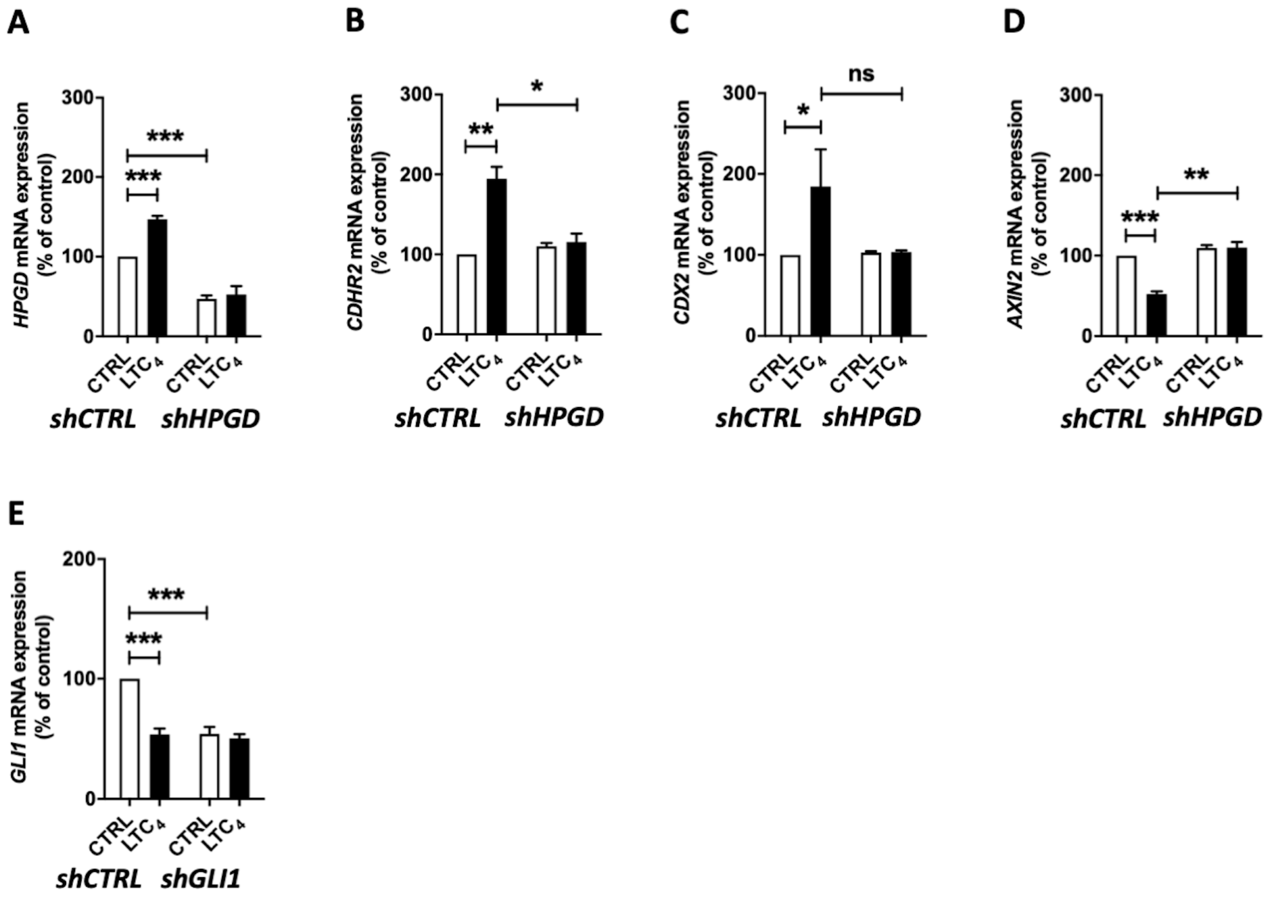

Supplementary Fig. S2

qRT-PCR analysis of **A**, *HPGD*, **B**, *CDX2*, **C**, *CDHR2*, and **D**, *AXIN2* gene expression in HT-29 cells transfected with control shRNA (*shCTRL*) or PGDH specific shRNA (*shHPGD*) followed by a 48 h simulation with LTC_4. **E**, qRT-PCR analysis of *GLI1* in HT-29 colon cancer cells transfected with *shCTRL* and *shGLI1* followed by 48 h stimulation with LTC_4. For qRT-PCR, *HPRT1* served as the housekeeping gene for normalization. Plots represent the mean ± SEM of data from 3-4 independent experiments, * P<0.05, ** P<0.01, *** P<0.001.
