# Supplementary material for: Tumour suppressor 15-hydroxyprostaglandin dehydrogenase induces differentiation in colon cancer via GLI1 inhibition
Source: Oncogenesis. 2020 Aug 19;9(8):74. doi: 10.1038/s41389-020-00256-0 (PMC7438320; doi:10.1038/s41389-020-00256-0)
Supplement: Supplementary file 4 — Supplementary Figure S3 [file 41389_2020_256_MOESM4_ESM.pdf]

Supplementary Fig. S3

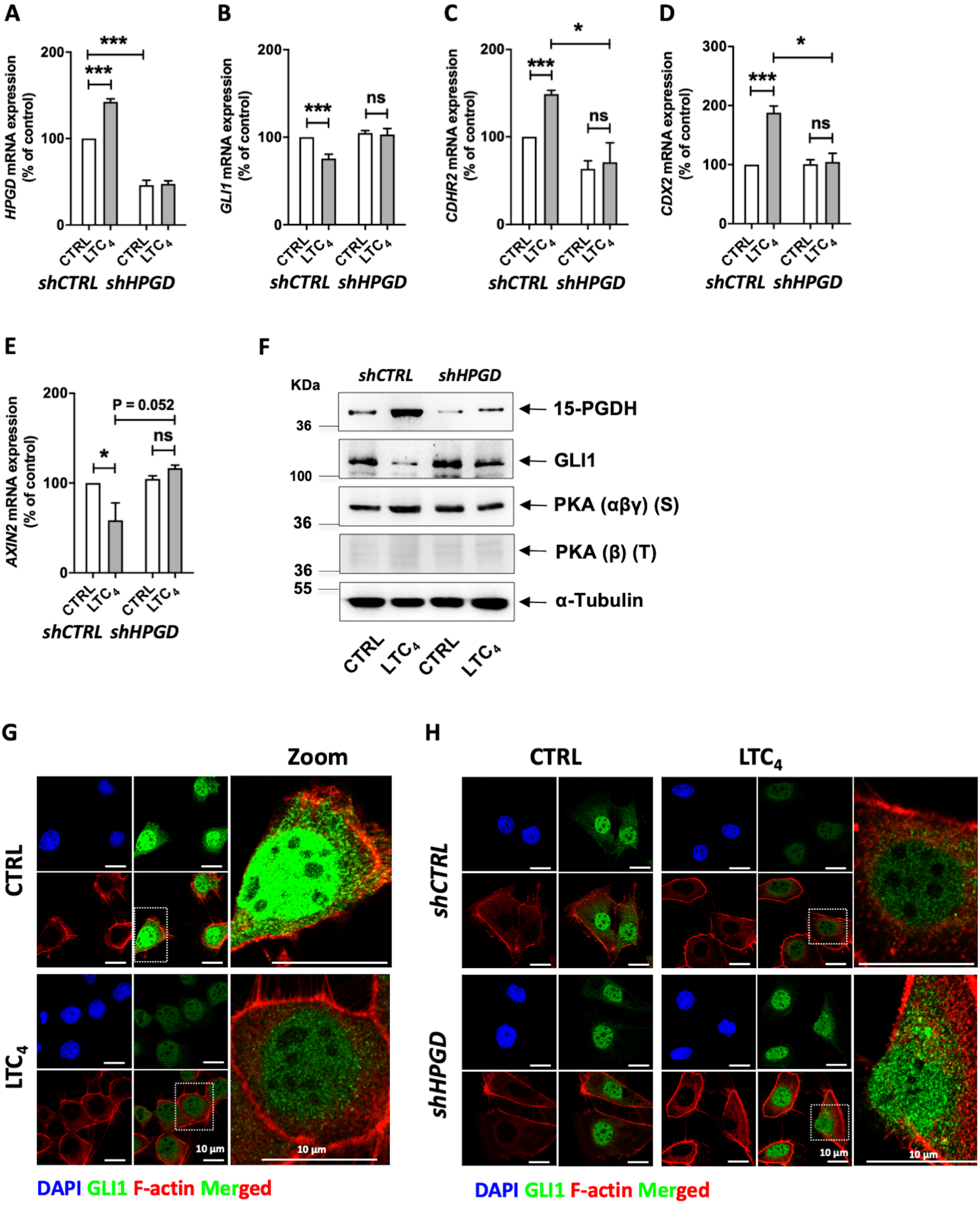

Supplementary Fig. S3

qRT-PCR validation of gene expression in *shCTRL*- and *shHPGD*-transfected Caco-2 colon cancer cells following LTC_4 stimulation for 48 h and compared with the unstimulated control. Markers of **A**, tumour suppression *15-PGDH* and **B**, *GLI1***,** **C**, differentiation regulation (*CDX2*), **D**, differentiation (*CDHR2*), and **E**, Wnt activation (*AXIN2*) compared between the *shCTRL* and *shHPGD* groups. *HPRT1* was used as the housekeeping gene for normalization. **F**, Immunoblotting analysis of whole-cell lysates of Caco-2 cells for 15-PGDH, GLI1 and PKA (αβγ subunit and β subunit) expression and **G**, immunofluorescence analysis of GLI1 in unstimulated and LTC_4-stimulated Caco-2 cells. **H**, Immunofluorescence analysis of GLI1 in unstimulated and LTC_4-stimulated Caco-2 cells and compared between the *shCTRL*- and *shHPGD*-transfected groups. α-Tubulin served as the loading control. Plots represent the mean ± SEM of data from 3-4 independent experiments, * $P<0.05$, *** $P<0.001$.
