# Supplementary material for: Tumour suppressor 15-hydroxyprostaglandin dehydrogenase induces differentiation in colon cancer via GLI1 inhibition
Source: Oncogenesis. 2020 Aug 19;9(8):74. doi: 10.1038/s41389-020-00256-0 (PMC7438320; doi:10.1038/s41389-020-00256-0)
Supplement: Supplementary file 5 — Supplementary Figure S4 [file 41389_2020_256_MOESM5_ESM.pdf]

Supplementary Fig. S4

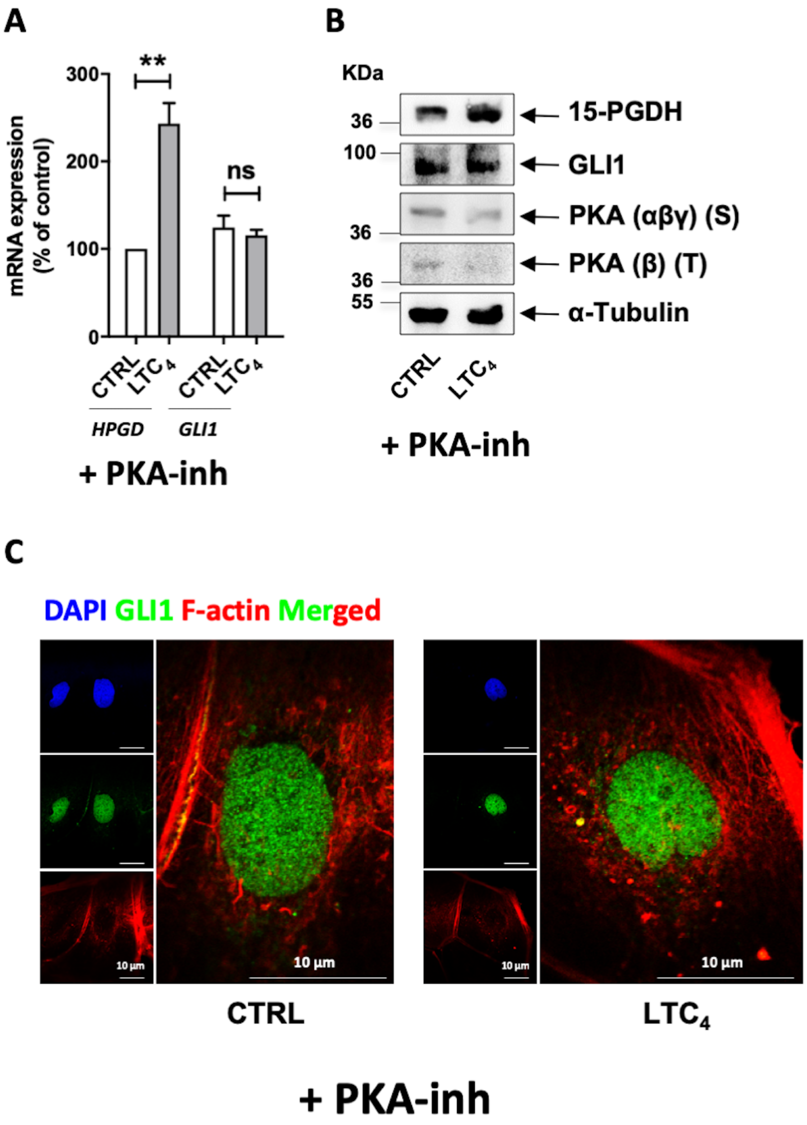

Supplementary Fig. S4

A, qRT-PCR analysis of 15-PGDH and GLI1 in Caco-2 cells treated with the PKA inhibitor H89 (PKA-inh) for 6 h followed by LTC_4 for 48 h. **B**, Western blot analysis showing expression of 15-PGDH, GLI1, phospho-PKA (αβγ subunit and β subunit) in Caco-2 cells treated with the PKA inhibitor H89 (PKA-inh) for 6 h followed by LTC_4 for 48 h. **C**, Immunofluorescence analysis of GLI1 in Caco-2 cells treated with the PKA inhibitor H89 (PKA-inh) for 6 h followed by LTC_4 for 48 h. *HPRT1* was used as the housekeeping gene for normalization of the qRT-PCR gene expression data. Plots represent the mean ± SEM of data from 3-4 independent experiments, ** $P < 0.01$.
