# Supplementary material for: Tumour suppressor 15-hydroxyprostaglandin dehydrogenase induces differentiation in colon cancer via GLI1 inhibition
Source: Oncogenesis. 2020 Aug 19;9(8):74. doi: 10.1038/s41389-020-00256-0 (PMC7438320; doi:10.1038/s41389-020-00256-0)
Supplement: Supplementary file 6 — Supplementary Figure S5 [file 41389_2020_256_MOESM6_ESM.pdf]

Supplementary Fig. S5

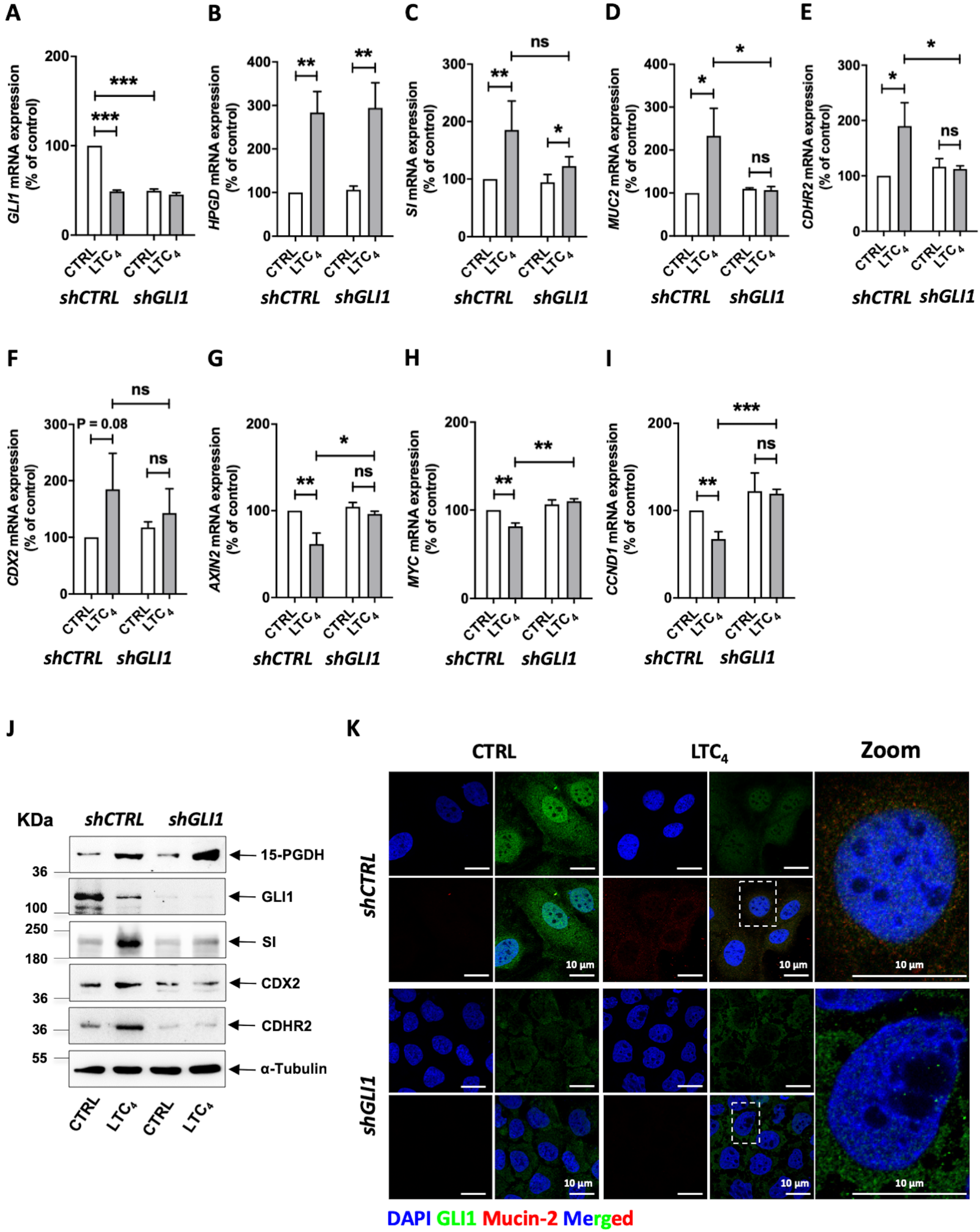

Supplementary Fig. S5

Caco-2 colon cancer cells were transfected with *shCTRL* or *shGLI1* followed by LTC_4 stimulation for 48 h. q-RT-PCR analysis of **A**, *HPGD*, **B**, *GLI1*, **C**, *SI*, **D**, *MUC2*, **E**, *CDHR2*, **F**, *CDX2*, **G**, *AXIN2*, **H**, *MYC*, and **I**, *CCND1*. **J**, Western blot analysis of whole-cell lysates for 15-PGDH, GLI1, SI, CDX2, and CDHR2 expression and **K**, immunofluorescence analysis of GLI1 and Mucin-2 in unstimulated and LTC_4-stimulated cells and compared between the *shCTRL* and *shGLI1* groups. α-Tubulin served as the loading control for Western blot analysis. For qRT-PCR, *HPRT1* was used as the housekeeping gene for normalization. Data are represented as the mean ± SEM of data from 3 independent experiments, * $P<0.05$, ** $P<0.01$, *** $P<0.001$.
