# Supplementary material for: Tumour suppressor 15-hydroxyprostaglandin dehydrogenase induces differentiation in colon cancer via GLI1 inhibition
Source: Oncogenesis. 2020 Aug 19;9(8):74. doi: 10.1038/s41389-020-00256-0 (PMC7438320; doi:10.1038/s41389-020-00256-0)
Supplement: Supplementary file 7 — Supplementary Figure S6 [file 41389_2020_256_MOESM7_ESM.pdf]

# Supplementary Fig. S6

## HT-29

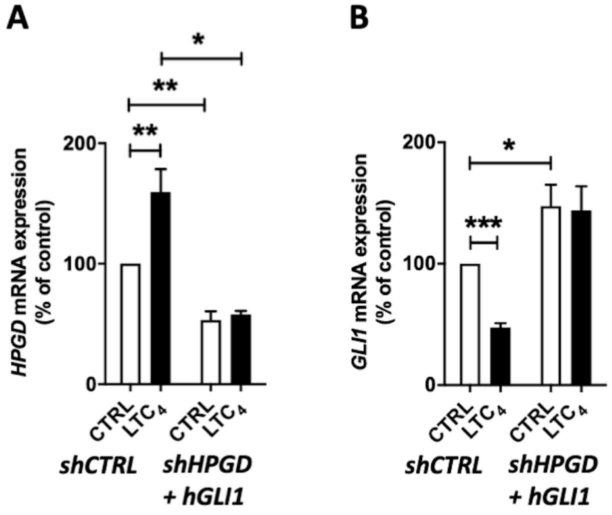


**Supplementary Fig. S6**

HT-29 cells were transfected with shCTRL and co-transfected with shHPGD and hGLI1 followed by LTC4 stimulation for 48 h. Graphs showing qRT-PCR analysis of **A,** HPGD and **B,** GLI1. For qRT-PCR, HPRT1 served as the housekeeping gene for normalization. Graphs represent data from 3-4 independent experiments and represent the mean ± SEM, * P<0.05, ** P<0.01, *** P<0.001.
