# Supplementary material for: Tumour suppressor 15-hydroxyprostaglandin dehydrogenase induces differentiation in colon cancer via GLI1 inhibition
Source: Oncogenesis. 2020 Aug 19;9(8):74. doi: 10.1038/s41389-020-00256-0 (PMC7438320; doi:10.1038/s41389-020-00256-0)
Supplement: Supplementary file 8 — Supplementary Figure S7 [file 41389_2020_256_MOESM8_ESM.pdf]

Supplementary Fig. S7

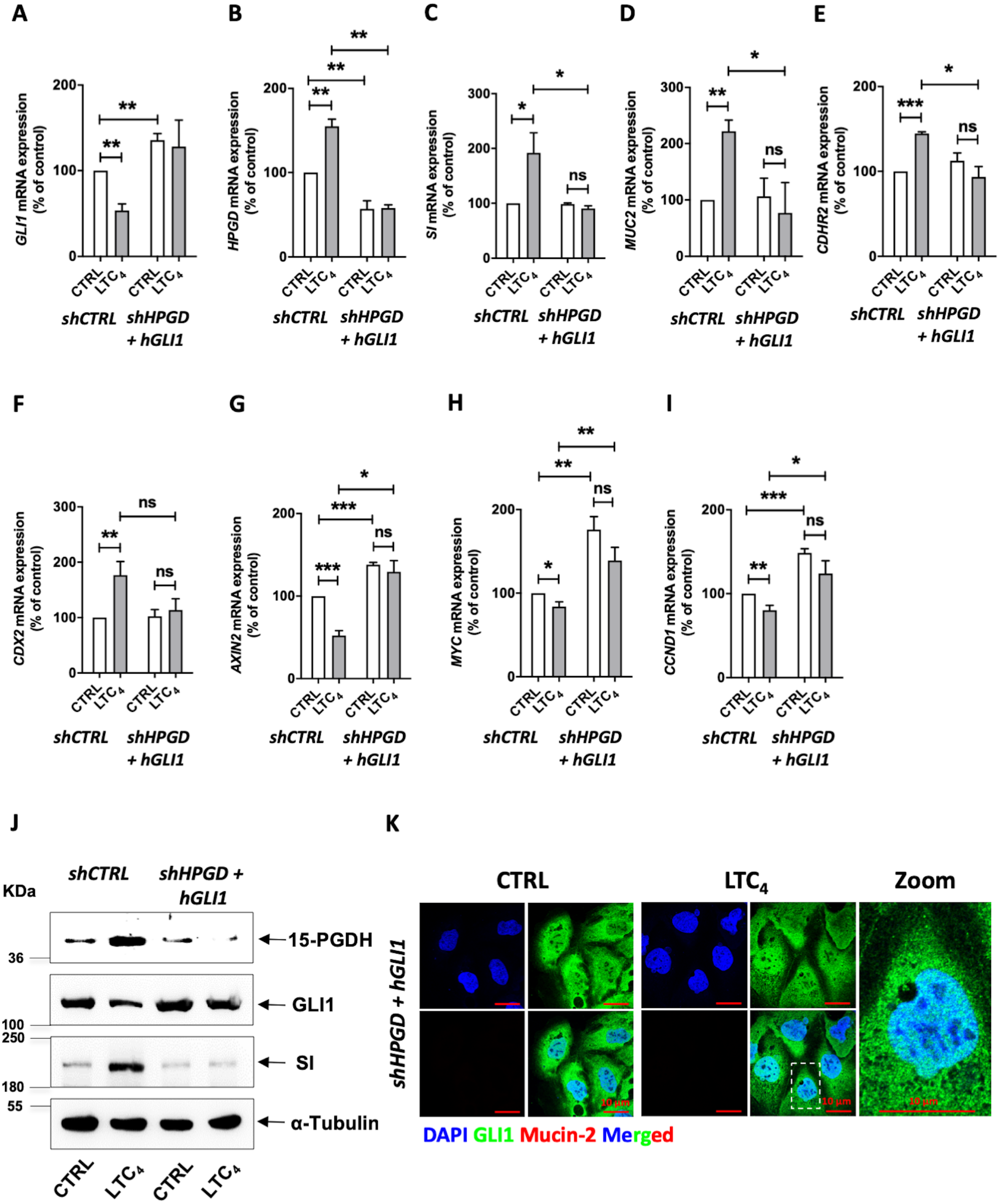

Supplementary Fig. S7

Caco-2 cells were transfected with *shCTRL* and co-transfected with *shHPGD* and *hGLI1* followed by LTC_4 stimulation for 48 h. Graphs showing qRT-PCR analysis of **A**, *HPGD*, **B**, *GLI1*, **C**, *SI*, **D**, *MUC*2, **E**, *CDHR2*, **F**, *CDX2*, **G**, *AXIN2*, **H**, *MYC*, and **I**, *CCND1*. **J**, Western blot analysis of whole-cell lysates for 15-PGDH, GLI1 and SI expression and **K**, immunofluorescence analysis of GLI1 and Mucin-2 in unstimulated and LTC_4-stimulated cells and compared between the *shCTRL* and *shHPGD* + *hGLI1* groups. α-Tubulin served as the loading control for immunoblotting analysis. For qRT-PCR, *HPRT1* served as the housekeeping gene for normalization. Graphs represent data from 3-4 independent experiments and represent the mean ± SEM, * $P<0.05$, ** $P<0.01$, *** $P<0.001$.
