# Supplementary material for: Tumour suppressor 15-hydroxyprostaglandin dehydrogenase induces differentiation in colon cancer via GLI1 inhibition
Source: Oncogenesis. 2020 Aug 19;9(8):74. doi: 10.1038/s41389-020-00256-0 (PMC7438320; doi:10.1038/s41389-020-00256-0)
Supplement: Supplementary file 9 — Supplementary Figure S8 [file 41389_2020_256_MOESM9_ESM.pdf]

Supplementary Fig. S8

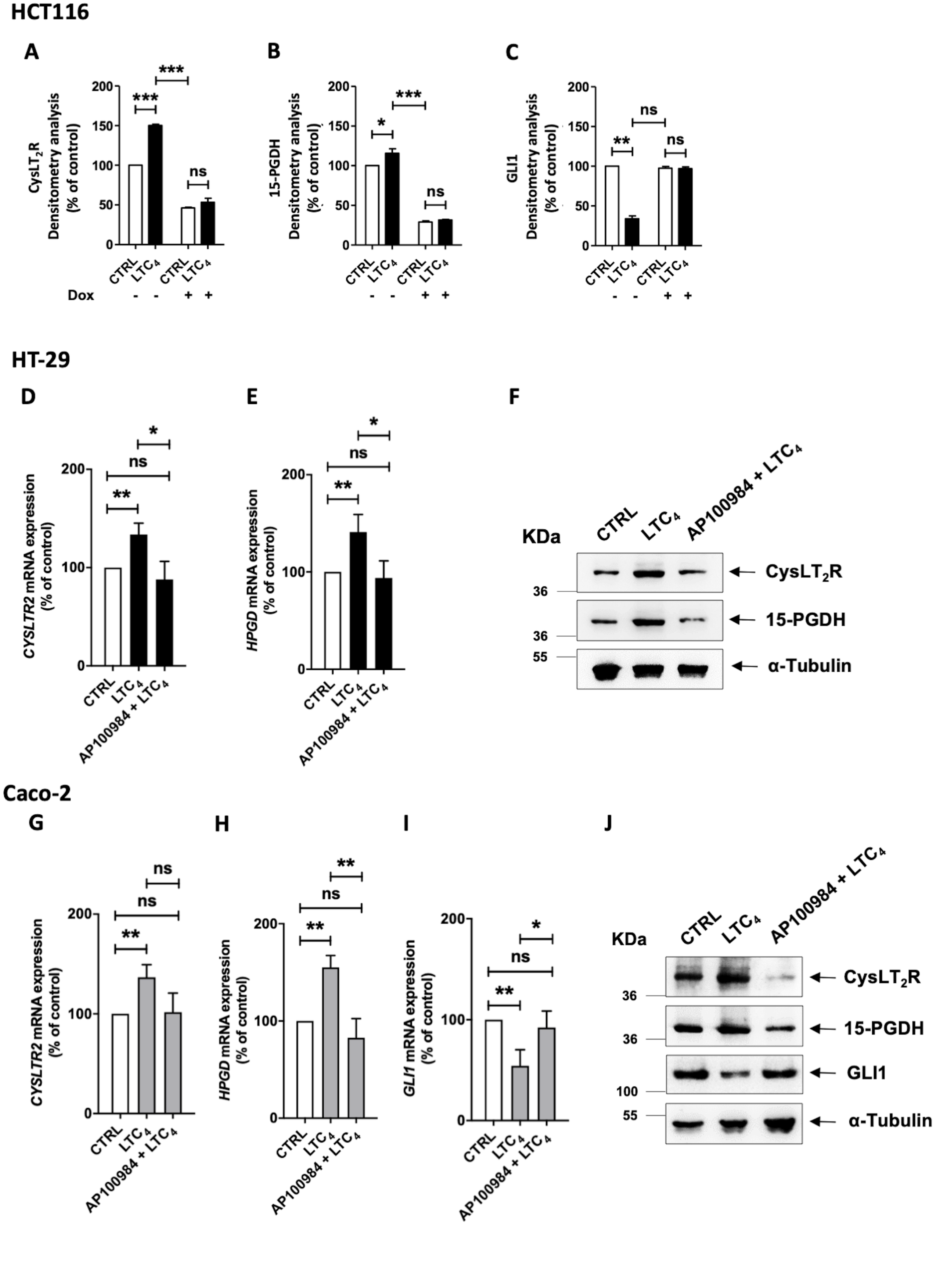

Supplementary Fig. S8

Densitometric analysis showing relative protein expression (% control) for **A**, CysLT$_2$R, **B**, 15-PGDH, and **C**, GLI1 in Dox-inducible HCT116 CC cells with or without LTC$_4$ stimulation. qRT-PCR analysis showing gene expression of **D**, *CYSLTR2* and **E**, *HPGD* and Western blot analysis showing protein expression of **F**, CysLT$_2$R and 15-PGDH in HT-29 cells treated with LTC$_4$ alone, AP100984 (CysLT$_2$R specific antagonist) alone or a combination of both.

Gene expression of **G**, *CYSLTR2* , **H**, *HPGD* and **I**, *GLI1* and Western blot analysis showing protein expression of **J**, CysLT$_2$R, 15-PGDH, and GLI1 in Caco-2 cells treated with LTC$_4$ alone, AP100984 (CysLT$_2$R specific antagonist) alone or a combination of both. *HPRT1* was used as the housekeeping gene for normalization. α-Tubulin served as the loading control in the Wester blot assay. Data represent the mean ± SEM from 4-5 independent experiments, * $P<0.05$, ** $P<0.01$, *** $P<0.001$.
