# Supplementary material for: Tumour suppressor 15-hydroxyprostaglandin dehydrogenase induces differentiation in colon cancer via GLI1 inhibition
Source: Oncogenesis. 2020 Aug 19;9(8):74. doi: 10.1038/s41389-020-00256-0 (PMC7438320; doi:10.1038/s41389-020-00256-0)
Supplement: Supplementary file 10 — Supplementary Figure S9 [file 41389_2020_256_MOESM10_ESM.pdf]

Supplementary Fig. S9

HT-29

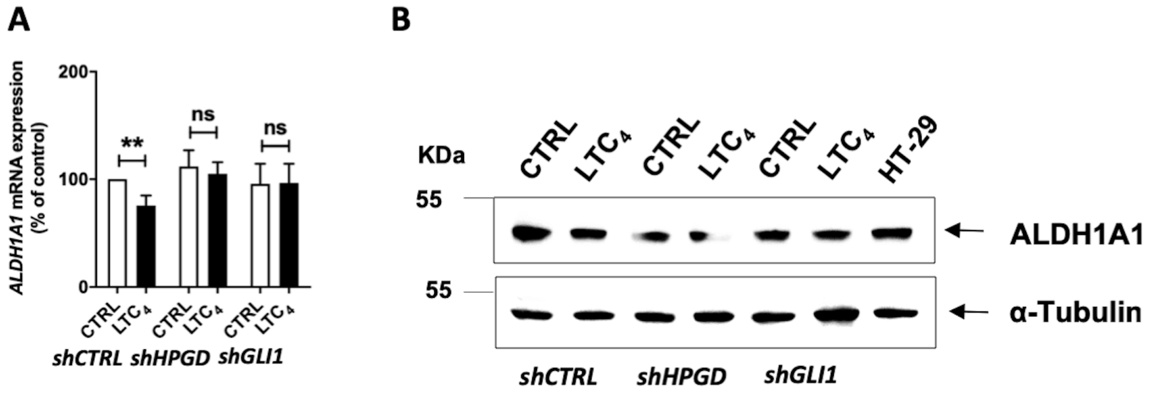

Caco-2

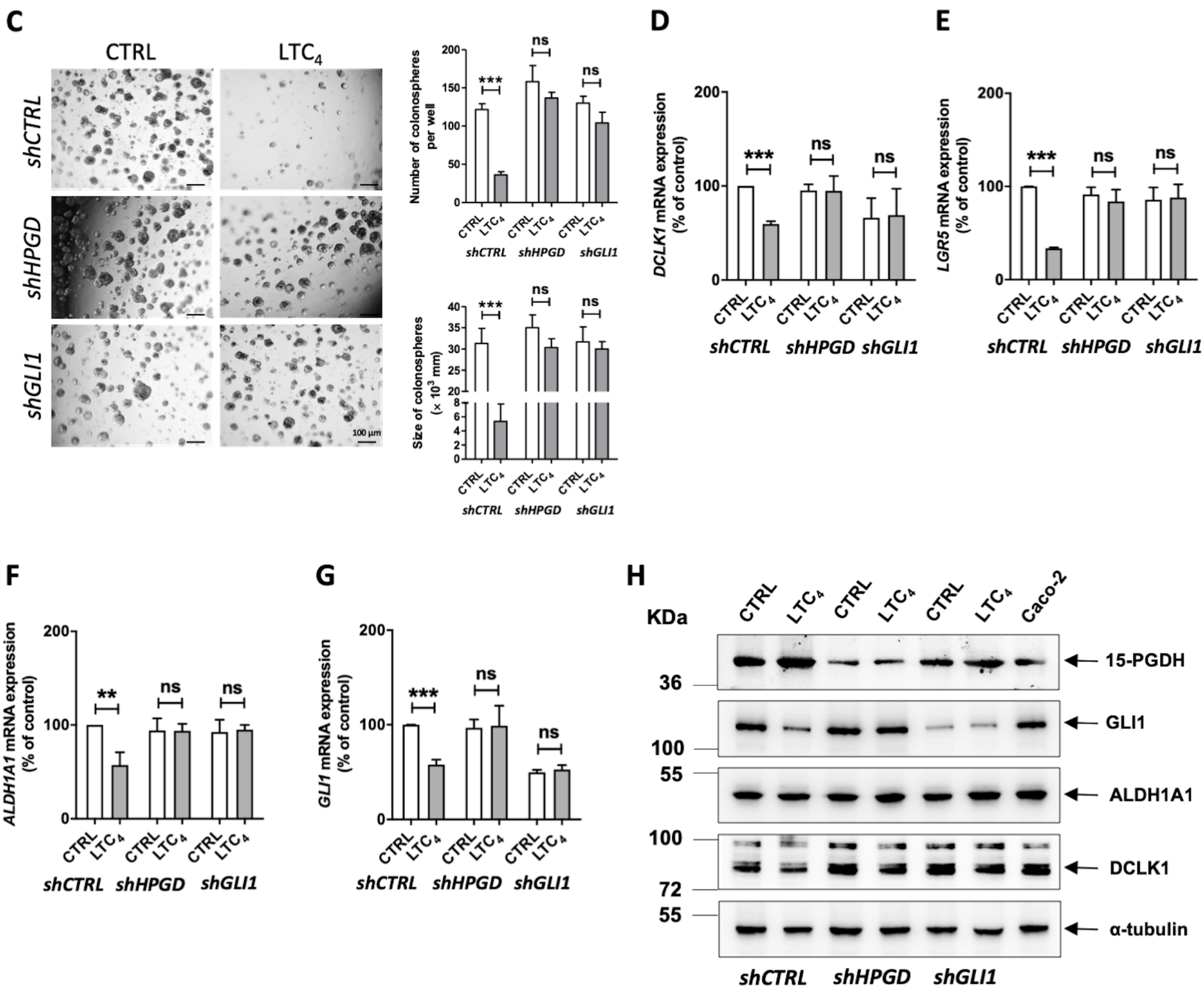

Supplementary Fig. S9

q-RT-PCR **A**, and Western blot analysis **B**, of the stemness marker *ALDH1A1* in *shCTRL-*, *shHPGD-* and *shGLI1*-transfected HT-29 cell-derived colonospheres after LTC_4 stimulation for 48 h. **C**, Representative images of colonospheres from Caco-2 cells transfected with *shCTRL*, *shHPGD* or *shGLI1* and further stimulated with LTC_4. Bar graphs showing the number of colonospheres formed per well and the effect on the size of colonospheres with or without LTC_4 stimulation and compared between the *shCTRL-*, *shHPGD-* and *shGLI1*-transfected groups. The q-RT-PCR analysis of **D**, *DCLK1*, **E**, *LGR5*, **F**, *ALDH1A1*, and **G**, *GLI1*. **H**, Western blot analysis showing the expression of 15-PGDH, GLI1, ALDH1A1, and DCLK1 in *shCTRL-*, *shHPGD-* and *shGLI1*-transfected Caco-2 cell-derived colonospheres after LTC_4 stimulation for 48 h. α-Tubulin served as the loading control. For qRT-PCR, *HPRT1* was used as the housekeeping gene for normalization. Data represent the mean ± SEM from 4-5 independent experiments, ** $P<0.01$, *** $P<0.001$.
